# Digital health technologies for peripartum depression management among low-socioeconomic populations: perspectives from patients, providers, and social media channels

**DOI:** 10.1186/s12884-023-05729-9

**Published:** 2023-06-03

**Authors:** Alexandra Zingg, Tavleen Singh, Amy Franklin, Angela Ross, Sudhakar Selvaraj, Jerrie Refuerzo, Sahiti Myneni

**Affiliations:** 1 McWilliams School of Biomedical Informatics, University of Texas Health Science Center at Houston, Houston, TX USA; 2 Faillace Department of Psychiatry and Behavioral Sciences, University of Texas Health Science Center at Houston, McGovern Medical School, Houston, TX USA; 3 UT Physician’s Women’s Center, University of Texas Health Science Center at Houston, Houston, TX USA

**Keywords:** Mental health, Digital health, Mobile health, Social media

## Abstract

**Background:**

Peripartum Depression (PPD) affects approximately 10–15% of perinatal women in the U.S., with those of low socioeconomic status (low-SES) more likely to develop symptoms. Multilevel treatment barriers including social stigma and not having appropriate access to mental health resources have played a major role in PPD-related disparities. Emerging advances in digital technologies and analytics provide opportunities to identify and address access barriers, knowledge gaps, and engagement issues. However, most market solutions for PPD prevention and management are produced generically without considering the specialized needs of low-SES populations. In this study, we examine and portray the information and technology needs of low-SES women by considering their unique perspectives and providers’ current experiences. We supplement our understanding of women’s needs by harvesting online social discourse in PPD-related forums, which we identify as valuable information resources among these populations.

**Methods:**

We conducted (a) 2 focus groups (n = 9), (b) semi-structured interviews with care providers (n = 9) and low SES women (n = 10), and (c) secondary analysis of online messages (n = 1,424). Qualitative data were inductively analyzed using a grounded theory approach.

**Results:**

A total of 134 open concepts resulted from patient interviews, 185 from provider interviews, and 106 from focus groups. These revealed six core themes for PPD management, including “Use of Technology/Features”, “Access to Care”, and “Pregnancy Education”. Our social media analysis revealed six PPD topics of importance in online messages, including “Physical and Mental Health” (n = 725 messages), and “Social Support” (n = 674).

**Conclusion:**

Our data triangulation allowed us to analyze PPD information and technology needs at different levels of granularity. Differences between patients and providers included a focus from providers on needing better support from administrative staff, as well as better PPD clinical decision support. Our results can inform future research and development efforts to address PPD health disparities.

**Supplementary Information:**

The online version contains supplementary material available at 10.1186/s12884-023-05729-9.

## Background

Peripartum depression (PPD) is diagnosed as a major depressive episode occurring at any time during pregnancy or shortly after childbirth [1]. Symptoms are similar to general depression, but women experiencing PPD can also exhibit symptoms specific to pregnancy and childbirth. Examples are negative thoughts and feelings regarding motherhood and detachment from the infant [2]. Symptoms are also much more severe and long-lasting than those of the common and transient “baby blues”. PPD affects approximately 10% of new mothers and pregnant women in the U.S. annually, representing a significant population [3, 4]. However, many women do not receive the necessary treatment for PPD because of barriers at the individual, social, and healthcare system levels. Such barriers include reluctance to disclose mental health struggles to family members or caregivers, social stigmas, misguided expectations on the role of motherhood, and not having appropriate PPD knowledge or access to mental health resources [5].

Several risk factors are associated with perinatal depression such as a history of depression, stressful life events, physical or sexual abuse, unplanned or unwanted pregnancy, inadequate social and financial support, intimate partner violence, co-morbid medical conditions, and complications during pregnancy [6]. Certain groups of women are at higher risk for developing PPD. These include women of low socioeconomic status (low-SES) [7], immigrant women [8], and women with high-risk pregnancies [9]. Additionally, health disparities have been reported in the areas of PPD literacy and access to care. Perinatal women with low education and income tend to have limited knowledge about depression and available courses of treatment, significantly reducing their ability to recognize symptoms correctly and seek help [10]. Similarly, in the U.S., low-income women eligible for Medicaid insurance experience greater difficulty than those with private insurance in receiving care from mental health specialists such as psychiatrists [11].

Digital health technologies are seen as a viable pathway to address the disparities mentioned above in PPD care, and digital interventions aimed at managing PPD have employed formats such as text messaging [12–15], online delivery of therapy programs [16–21], and mHealth apps [22–25]. Each of these formats are shown to be effective in different areas of PPD management. For example, text messaging has been suggested as a feasible method for PPD screening [14, 15], and also, online delivery of cognitive behavioral therapy has successfully helped perinatal women reduce depression symptoms [20]. In addition, women very frequently use other digital media such as online social forums to obtain information on different aspects of their pregnancy [26], including the mental health aspect [27, 28]. Research studies suggest that the anonymous nature and reduced stigma of the online environment allow women to feel more comfortable disclosing mental health struggles and adverse pregnancy experiences [29, 30]. Prabhakar and colleagues [31] propose that social media channels frequently act as important social support networks for perinatal women when they do not receive adequate support from other sources such as their partners, family, and friends. This was especially true for women seeking information on practical topics such as breastfeeding. A possible underlying mechanism through which these platforms support women is the exchange of personal experiences, information, and advice during the pregnancy journey [32–34].

While there have been recent advances in digital health for PPD management, most digital health market solutions currently available are developed in a one-size-fits-all manner [35]. One evidence-based intervention for PPD management specifically designed for low-SES women is the Mother and Babies (MB) course, originally designed for in-person prevention of PPD. Gewali and colleagues [36] adapted the program to be delivered as a social media group intervention targeting perinatal women between 14 and 24 years old. Results showed that participants desired the web-based version of MB to include additional components such as peer support channels and the ability to ask medical questions. Barrera and colleagues have also made efforts to adapt the MB course into an electronic format and add text message reminders [37, 38], with results suggesting benefits of the intervention to perinatal women of racially and ethnically diverse backgrounds. Other digital health interventions directed at low-SES women have included successfully answering health questions through two-way text messaging [39], and supportive text messages as a supplement to traditional counseling [15]. Most studies targeting PPD management among low-SES perinatal women focus on feasibility trials and gathering of perspectives from individual patients, however very few extend such efforts by also considering perspectives from sources such as providers of low-SES perinatal women or social media content in PPD-specific online forums. This represents a missed opportunity for enriching current insights into this specific population’s information and technology needs. For example, in 2021 social media sites were being used by 69% of all adults in U.S. households earning less than $30,000, and they were being used by 78% of all women in the U.S. [40].

In order to improve our current understanding of how PPD digital health technologies can be made more suitable to a low-SES population, in this study we elaborate on our analysis of stakeholder perspectives ranging from in-depth interviews to large-scale social media content analysis of PPD-specific online forums. Such analysis allows us to discover multilevel insights including personal opinions as well as communication topics of importance to a broader sample of perinatal women. Our aim is to address existing health disparities in peripartum depression by engaging key stakeholders to identify optimal digital engagement pathways.

## Methods

### Study design

**Fig. 1:**
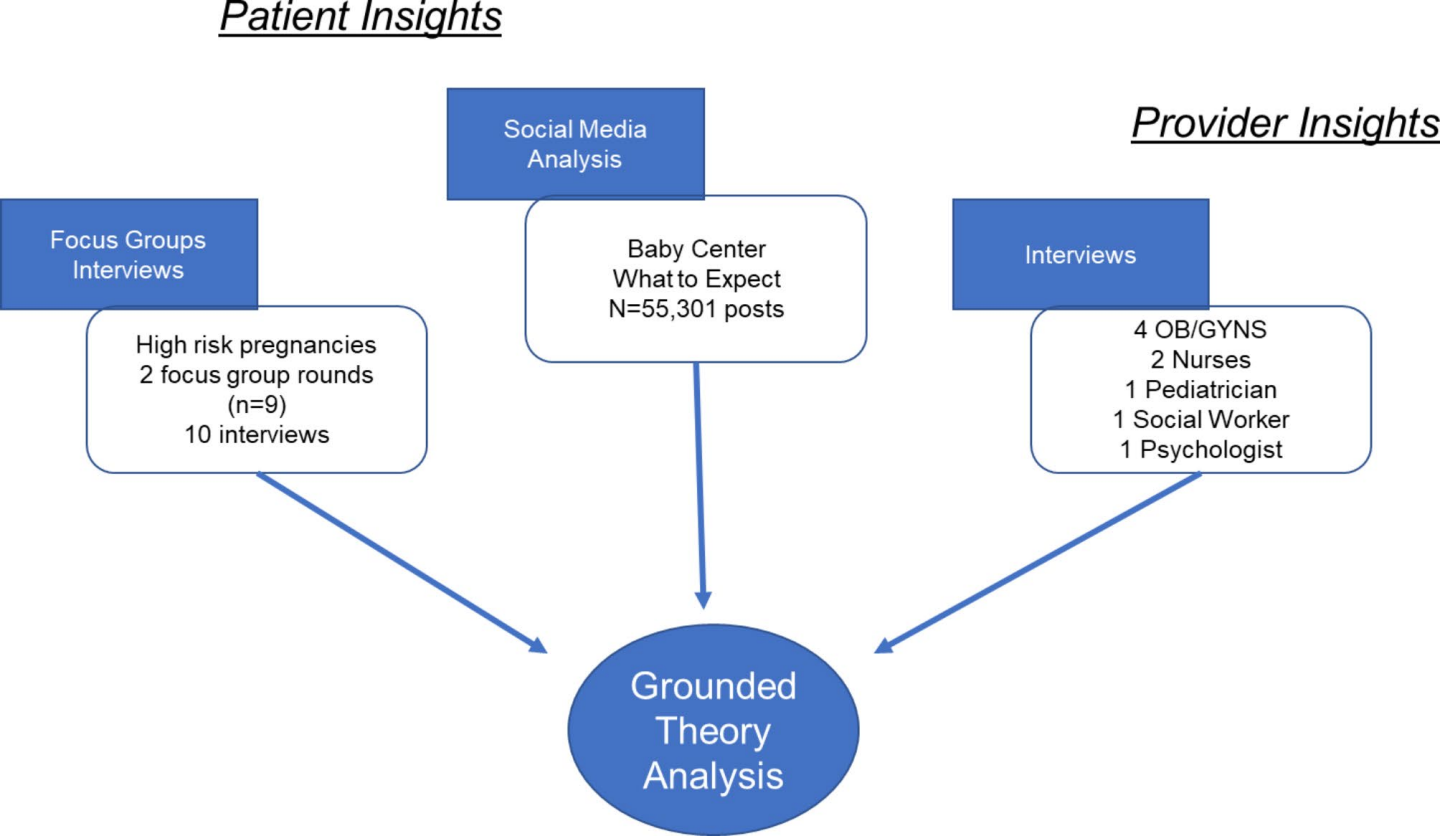
Research methodology for assessment of PPD information and technology needs

In this study, we describe our qualitative analysis of perspectives from low-SES women and their perinatal care providers, gathered through a triangulation of data sources. Our first data point consists of patient insights from focus groups and interviews with low-SES perinatal patients. For our second data point, we gather perspectives from interviews with their care providers. Our third data point consists of content analysis of PPD-specific online messages, which we use to supplement our focus groups and interviews. Our data collection and analysis methodology are illustrated in Fig. 1. This figure highlights the triangular nature of our study, where our three data sources (focus groups and interviews with patients, social media analysis, and provider interviews) inform the grounded theory approach through which we extract information and technology needs.

### Capturing patient insights

- Focus Groups and Interviews.

We have individually interviewed 10 patients and held two focus group sessions with 9 patients [41, 42]. Data collection occurred at the UT Physicians Maternal and Fetal Medicine (MFM) clinic in Houston, Texas. The clinic treats high-risk pregnancy conditions including fetal congenital anomalies and placental disorders. In order to participate, patients had to be at least 18 years old and seeking care at the clinic. Due to English being the most common language spoken by the Houston population [43], we focused on recruiting English-speaking patients for this study. Prior to participating in focus groups or interviews, patients completed a survey capturing characteristics including demographics, pregnancy, and experiences with PPD (Appendix A). Patients were not excluded based on income, and we have followed the U.S. Department of Housing and Urban Development adjusted home income limits for Texas to determine the socioeconomic status of participants’ households. These guidelines classify households according to number of people living in the household and total household annual income [44].

Focus groups were led by author SM and interviews by author AZ. Author SM has credentials of a Ph.D. in Health Informatics and is an Associate Professor at the McWilliams School of Biomedical Informatics at UTHealth and Associate Director of its Center for Digital Health and Analytics. Her areas of expertise include social phenotyping and design and development of digital health interventions focused on addressing health disparities. Author AZ is a recent graduate of the Ph.D. in Health Informatics program at the McWilliams School of Biomedical Informatics. Her training and credentials also include a Masters of Public Health and Masters of Science in Health Informatics. At the time of the study, her occupation was Graduate Research Assistant under supervision of author SM. Participants had no prior relationship with the authors, and during the focus groups and interviews they were introduced to the authors’ credentials and reasons for the study.

We followed a semi-structured approach to focus groups and interviews, where we used question guides to help carry forward the discussion. Examples of questions used are: “Do you like using health apps to get answers to questions, or would you rather sit down with your doctor when you have a question?”, “What kind of information do you actually get from these apps?” (Appendix B). While our interview guides included additional questions inviting patients to discuss their preferred design choices for PPD digital health technologies, in this study we focus on analyzing patient answers to questions regarding their mental health information-seeking behavior and current use of health technologies in the management of their pregnancy. Focus groups and interviews were audio recorded and transcribed verbatim.

b) Social Media.

Based on input from our focus group and interview participants that suggested their active involvement in social media channels, we have analyzed PPD-specific online social forums [41, 45]. Our participants specifically mentioned their frequent use of the pregnancy apps What to Expect [46] and Baby Center [47], two of the most popular pregnancy apps used by women in the U.S. and internationally. Therefore, our social media dataset was extracted from the forums “Postpartum Depression” from What to Expect and “Postpartum Depression, Anxiety, and Related Topics” from Baby Center. Posts were extracted using the software Scrapy [48], a Python-based library for web crawling purposes. We obtained a total of 12,416 post threads and 55,301 posts exchanged by 9,364 individuals. Our dataset spanned from years 2008 to 2022. Our selected social forums and their respective posts are open to the public. We have additionally removed any potential identifiers of usernames from our dataset.

### Capturing provider insights

Nine perinatal healthcare providers practicing in the Texas Medical Center were interviewed. To be included in our study, providers had to be practicing in a clinic where women at risk for or diagnosed with PPD can receive treatment. We also included providers who treated children whose parents might be at risk for PPD (i.e., a NICU pediatrician). Providers were recruited at various sites within the UT Physicians system. Provider interviews were approximately 30 min in length. Interviews were led by author AZ, and before the interviews providers were introduced to her credentials and the goals of the study. Providers had no prior established relationship with the author. Similar to patient interviews, question guides were used to facilitate discussion (Appendix C), and we have focused on analyzing provider responses to questions regarding their current management of PPD cases and the role that digital technologies have in their practice. An example question asked to providers is: “From your perspective, what are some difficulties that PPD health care team members face when trying to help perinatal women prevent or manage PPD?”. Provider interviews were also recorded and transcribed verbatim.

### Data analysis

- Focus Group and Interview Analysis.

Using Dedoose analysis software, all focus group and interview transcripts were coded using a grounded theory approach [49, 50], which allowed insights to emerge directly from the data. In this approach, the first step is open coding, whereby every comment is read line-by-line and essential concepts and ideas brought up by individuals are identified as they emerge from the data. Additionally, a process of constant comparison ensures code reliability and consistency [50, 51]. This iterative data analysis enabled us to refine and adapt our methodological frameworks based on emerging findings. This approach allowed us to gain understanding of the social and cultural context in which phenomena occur, which helped us uncover the meanings, perspectives, and experiences of women, providing rich insights into the context-specific factors that influence their behavior and decision-making. Such an inductive approach also minimizes researcher bias without imposing preconceived notions or theories and is optimal to extract the information and technology needs of vulnerable peripartum women, since it provides us with precise concepts and design and development insights that can be applied and examined in further studies, allowing for deeper exploration of interventions, programs, or strategies tailored to specific contexts for our target audience [52, 53].

In our study, open coding of patient and provider interviews was independently conducted by two researchers (AR and AZ). Open coding of focus group transcripts was also done independently by two researchers (AF and AZ). An example of this process for patient interviews is illustrated below, in which we analyze a comment where a patient describes how she uses a mobile app to manage her current pregnancy:

*“I get on it every other day, um, and definitely as my week changes for the pregnancy just because it gives you all the different information, you know, as the baby’s growing and things on the baby’s health and things that my body goes through as well.” (Participant I2)*.

The main open concepts extracted from this excerpt were: “pregnancy”, “information”, “baby”, and “health*”*. During open coding, a process of constant comparison was used to ensure consistency in code occurrences and the concepts they represented, enabling the formation of exact codes. Once no new open codes emerged from the data, a series of discussion meetings were held among coders to create a final list of codes and ensure researcher agreement in code application. Open coding was followed by the process of axial coding, an essential step in grounded theory analysis where the core themes of discussion emerge from assessing relationships and patterns among open codes. As an example of axial coding, the previously identified open codes of “pregnancy”, “information”, *“*baby”, and *“*health” were grouped into the core theme of “Pregnancy Education”. Grouping of open codes to form axial codes was done iteratively among all coders. Once no new axial codes emerged, indicating thematic saturation, a final list of core themes was agreed upon.

b) Social Media Analysis.

A total of 1424 posts were randomly selected from our social media dataset for manual coding. We discovered major information and technology themes discussed by participants in PPD online social forums by employing the previously described grounded theory approach. Our manual coding is multi-label, where one post can fall under one or more themes. Consistency and agreement in resulting themes were ensured by having one coder (AZ) manually code all 1424 posts, and a second coder (TS) manually code a subset of 150 randomly selected posts. The measure of Cohen’s Kappa was used to quantify interrater reliability, and an iterative process of discussions was used to resolve disagreements among raters. Interrater reliability was consistently substantial across all themes, with the highest Cohen’s Kappa measure of 1.00 in the theme of “Family and Friends”, followed by “Mother and Infant Dyad” (k = 0.98), and “Doctor and Patient Dyad” (k = 0.92) (Table 1).

**Table 1:** Interrater Reliability Among PPD Social Media Themes

Theme	Cohen’s Kappa
Medications	0.84
Family and Friends	1.00
Physical and Mental Health	0.85
Social Support	0.84
Mother and Infant Dyad	0.98
Doctor and Patient Dyad	0.92

## Results

### Patient and provider characteristics

All of our focus group and interview patient participants report being pregnant. The majority (n = 10) fall in the age range of 25–34 years old, n = 5 between 18 and 24 years old, and n = 4 between 35 and 44 years old. In total, 15 of 19 participants (78.9%) fell within the age range of 18–34 years old. Most patients identify as Hispanic (n = 7), followed by Black (n = 6), White (n = 4), and Other (n = 1). The majority have an education level of some college (n = 5) or an associate’s degree (n = 5) and a household income of less than $40,000 (n = 14). In total, 15 of 19 participants (78.9%) lived in low-income households. Of nineteen patients, 13 report experiencing depression symptoms during their current pregnancy, and seven of these had mentioned it to their doctor. However, none of our patients have a depression diagnosis. Most patients look for information about pregnancy on the internet (n = 17), while some also consult other sources such as books, friends, families, and their doctors (n = 11) [54]. Our provider sample consists of OB/GYNs (n = 4), Neonatologist (n = 1), Nurses (n = 2), Psychologist (n = 1), and Social Worker (n = 1). All providers are female.

### Interview themes

A total of five common core themes are formed from patient and provider interviews, based on 134 open codes from patient interview transcripts, and 185 open codes from provider interview transcripts: (1) Use of Technology/Features, (2) Access to Care, (3) Pregnancy Education, (4) Social and Community Support, and (5) Sources of information. These themes are defined in Table 2.

**Table 2:** Top Interview Themes (Patients and Providers)

Theme	Definition
Use of Technology/Features	Comments where the individual describes the health technology applications and features she uses and for what purposes (pregnancy, family), as well as technology/feature characteristics that compelled or prevented them from using the application (price, perceived value of the features). It includes instances when the individual mentions features that she currently lacks or needs in their perinatal care.
Access to Care	Comments related to improving healthcare access, including the ability to communicate with providers, self-monitoring, education, and making appointments.
Pregnancy Education	Individual describes the pregnancy information they receive or want/lack from pregnancy applications.
Social and Community Support	Individual describes how a social forum app would assist them in creating social capital (friendships, community resources).
Sources of Information: Family	Individual prefers to receive information from FAMILY over doctors, electronic sources, or printed sources.
Sources of Information: Doctor	Individual prefers to receive information from DOCTOR over family, electronics, or printed sources.

In addition to these common themes, a unique theme to patients is “Privacy and Security”, where the patient discusses their concerns about their personal information’s safety when using technology to share information about their pregnancy. Patients emphasize a desire for technologies that allow them to connect with peers while maintaining a high level of privacy and confidentiality. They are hesitant to use channels such as Facebook due to its being a highly public platform, with an example comment being:

*“I know you could probably get that [community] from Facebook, but I would rather not do it on Facebook because it’s a lot of people …”. (Participant I5)*

Two themes that are unique to providers are:

1. “Managerial and Administrative Support for Providers”, where providers express the need for better support from management and administrative level positions in terms of having adequate resources to carry out optimal care for PPD patients (documentation, follow-up, interdisciplinary collaboration, workflow). An example comment is related to providing support to patients who experienced miscarriages:

*“It [miscarriage] is an extremely common issue and I don’t believe we have sufficient [mental health] support services to offer the between one in three and one in five women. I’m not saying that it wouldn’t be beneficial, it certainly would, but I don’t think that’s something we’ve had the capacity for.” (Participant P2).*

2) “Peripartum Depression Clinical Decision Support”, where providers express the need for better clinical decision support systems, specifically definitions and clinical practice guidelines for PPD (includes screening process and leveraging the patient history and patient-generated data). An example comment:

*“There’s not a formal screening process that I’m aware of; that doesn’t mean it’s not happening because I do know every parent or mother who has a baby in the NICU does get visited by a social worker, and so it may actually be part of their intake. I just, honestly, don’t know too well.” (Participant P6)*

### Focus Group Themes

**Fig. 2:**
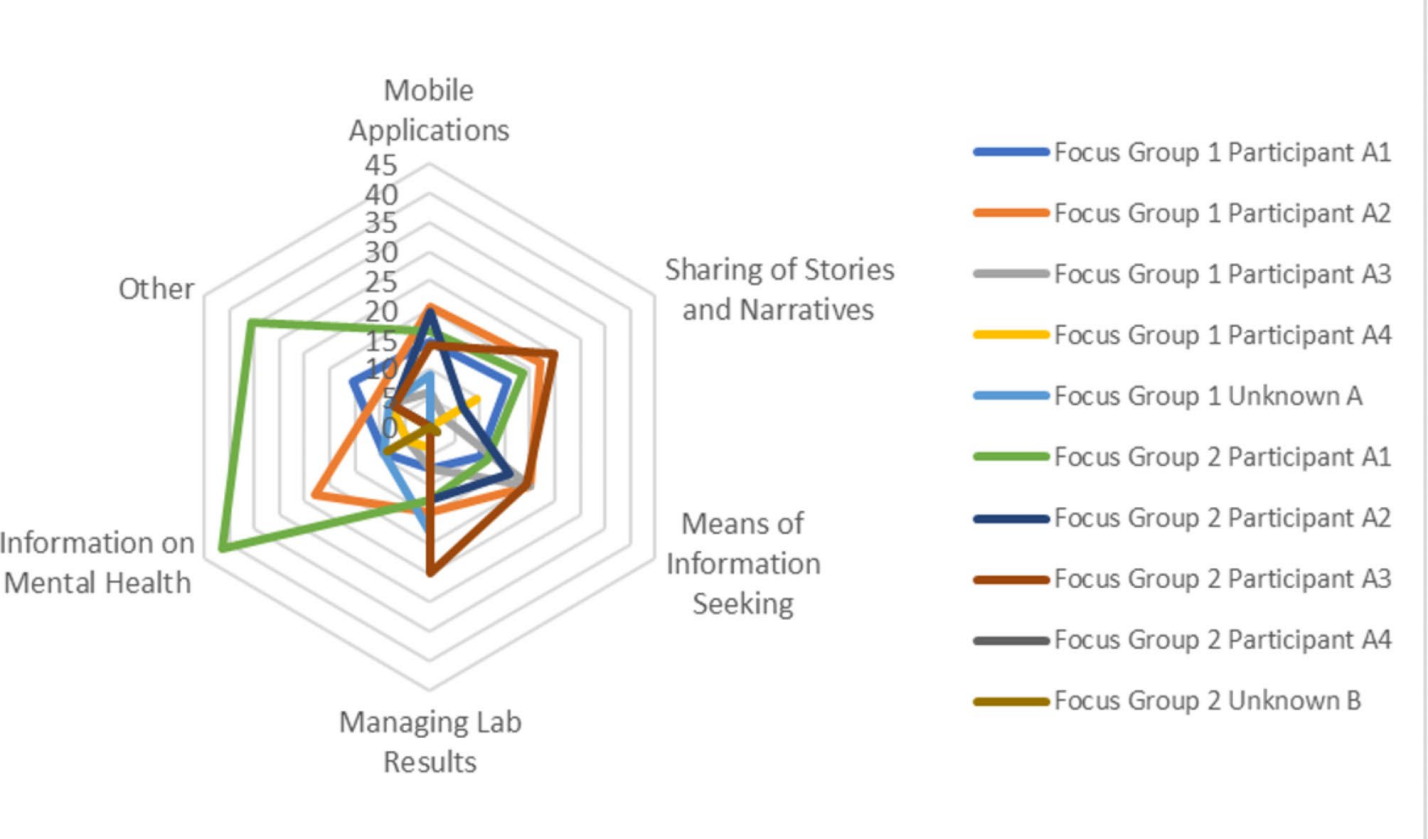
Distribution of PPD Information and Technology Themes across Focus Groups

A total of 106 open codes emerged from focus group transcripts, representing idea concepts ranging from “app use” to “family support”. For focus group session 1, the most active participant is participant A2 with 70 speaking turns, and the least active participant is participant A4 with 12 speaking turns. For focus group session 2, the most active participant is participant A2 with 72 speaking turns, and the least active participant is participant A3 with 42 speaking turns. Top open codes include: “Use of Mobile Applications”, “Sharing of Stories and Narratives”, “Information on Mental Health”, “Means of Information Seeking”, and “Managing Lab Results” (described in Table 3). Of these codes, “Use of Mobile Applications” is the one most mentioned in Focus Group 1 and “Means of Information Seeking” is the most mentioned in Focus Group 2. The distribution of codes is illustrated in Fig. 2. We have assigned these the core themes of “Use of Technology”, “Education/Information”, and “Digital Data” (Table 3).

**Table 3:** Top Focus Group Themes

Theme	Open Code	Definition		Sample comment	
Use of Technology	Use of Mobile Applications	Comments that describe how individuals navigate their pregnancy journey with the help of smartphone applications.		*“Well, for pregnancy, I use the What to Expect app. I use that one a lot. So, it gives you different stories of what you will go through, what symptoms you might have. It will also tell you what you need energy—or to eat. Things like that.” (Participant F2A2)*	
Education/ Information	Means of Information Seeking	Individuals describe how they use technologies such as social forums to gather information of interest about their pregnancy.		*“It was almost like, well, you don’t have a heartbeat so I asked other mothers, and they were, like, oh, that happened to me, just give it a couple of weeks and the heartbeat should be there then. Then a couple more weeks and the heartbeat is there.” (Participant F2A3)*	
	Information on Mental Health		Comments where individuals describe instances when they were wanting or needing information on mental health but could not find it	*“Of the seven apps that I have to track everything, nothing says, if you have a history of miscarriages—like I do—like both of us, I guess—or if you have a history of depression, anxiety, or PTSD, or all of these different things, how do you now cope with, is this a normal feeling?” (Participant F2A1)*	
	Sharing of Stories and Narratives		Individuals share pregnancy information and experiences through a personal story-telling format.	*“I’m in the Mommy group, so a lot of people who’s been out there that’s gotten pregnant, they still sit down and talk and say, okay, I’m going through this—because I was scared because they tell me my baby didn’t even have a heartbeat—they was, like, oh, just give it time, like a couple weeks… I was, like, five weeks so it was still early—and the doctor already telling me to get rid of it.” (Participant F2A3)*	
Digital Data	Managing lab results		Individuals express confusion and sometimes frustration with the various channels available to share lab results as well as the want of lab results even if normal.	*“Sometimes they call—only if it’s abnormal, but some of us, we just want to know, hey, like I have a bleeding disorder—well, what’s my level? What’s my level? Should I be doing.” (Participant F1A3)*	

### Social media analysis

There are six major themes that emerged from our qualitative analysis of 1424 social media posts, with the most common theme being “Physical and Mental Health” (n = 725 posts, 50.9%), followed by “Social Support” (n = 674, 47.3%), “Medications” (n = 612, 42.9%), “Mother and Infant Dyad” (n = 461, 32.3%), “Family and Friends” (n = 428, 30.1%), and “Doctor and Patient Dyad” (n = 423, 29.7%). A minor number of posts (n = 17, 1.2%) are omitted from our analysis due to content of a miscellaneous nature. Most posts exchanged in our selected PPD-specific online communities are focused on sharing of personal experiences with PPD, including struggles, success stories, and advise for day-to-day management of symptoms. The providing of sympathy and friendliness are also prominent characteristics of individuals’ posts, and are crucial to encouraging positive health behaviors such as communicating PPD symptoms to providers. Definitions and examples of our six major themes are provided below.

- Family and Friends: These are posts where the individual specifically mentions relationships with family and/or close friends as an important factor in their mental health state. Role of family and friends can be direct or undirect, and positive or negative. In the following example, an individual describes how she projects her worries about her ill child onto her significant other, which has negatively affected their relationship.

*“My boyfriend is amazing, but lately I’ve been so against him. I’ve been taking everything I feel out on him without even realizing it?? I’m afraid due to my son being ill it’s bringing on PPD. I’m scared about it and I would get so much comfort in knowing I’m not the only mum going through this.” (Post # 65)*

b) Medications: Individuals’ statements or queries about pharmacological treatments for peripartum depression. Characteristics of these posts include questions about optimal dosing and medication options. Other characteristics include queries about medication effects on breastfeeding and medication effects on baby. As an example, in the next post an individual is wondering about the safety of a newly prescribed medication and what her peer’s experiences have been.

*“I’m 34 weeks and my Psych doctor recently prescribed Buspar 5 mg for myself. I still have to get an okay from my OB just wondering if anyone else has taken this Med during pregnancy and what their experience with it was ??”. (Post # 197)*

c) Physical and Mental Health: Individual describes changes in their body or state of mind during the peripartum period. These can include symptoms of anxiety, insomnia, negative thoughts or feelings, happiness, and exhaustion. In this next example, an individual describes in detail the physical symptoms she has experienced as a consequence of PPD, her frustration at the fluctuation of the symptoms, and her difficulty accepting them.

“…*I’m 6 weeks postpartum as of today. Having a hard time. I have anxious spells- with severe symptoms where I feel like I’m in a dream, panicky, heart racing, thoughts that I know I don’t actually think. […] Sometimes I’ll go all or most day and feel asymptomatic of PPD and I’ll want so badly to believe it’s gone. But the symptoms return and I’m left heartbroken. […]This is so unfair*.” *(Post # 12)*

d) Mother and Infant Dyad: Posts where an individual describes their interactions and relationship with their infant, of both positive and negative nature. These may focus on breastfeeding and the bonding process. In the illustrating post, we find an individual who feels resentment toward the difficult task of caring for her infant.

*“Same boat! my baby is 3 weeks old in 2 days and I don’t feel like I love him at all. I’m already depressed from not sleeping and with breast feeding and feeling like I can’t do anything because as soon as I start he’s hungry and feeling resentment towards your new baby doesn’t help. Makes me feel like I’m already failing as a mom. You’re not alone! I’ve been told it’ll pass so hopefully it does for the both of us”. (Post # 673)*

e) Social Support: Content where an individual provides support to their peers, and can be of four types: emotional (words of encouragement and kindness), an appraisal (feedback on a situation), instrumental (a practical tool), and informational (personal experiences or educational resources). For example, in the next post an individual is trying to reshape her peer’s self-perception by normalizing feelings of inadequacy as a new mom.

*“You’re not lazy or incompetent. You miss that time, which is totally normal since your life just changed. But having a kid is a TON of work. It’s not being lazy if you’re not always up to the task.” (Post # 8)*

f) Doctor and Patient Dyad: Individual describes interactions with their care providers as part of their mental health management. Such posts may include instances of disclosing PPD symptoms to providers, providers managing PPD treatment, and managing appointments. In this next post, an individual finds relief in her doctor’s reaction to her delayed seeking of PPD care.

I just went to the dr for ppd yesterday and my girl will be 7 months this week. The dr took it very seriously and said it can take a good year for the chemicals in our brain and our hormones to find their normal again. It’s never too late to be brave enough to ask for help??

*(Post # 865).*

## Discussion

The main goals of this study are to: (a) add to existing knowledge on stakeholders’ perspectives (patients, providers) regarding use of digital health technologies for PPD prevention and management, specifically among vulnerable populations, and (b) to provide insights to guide intervention design and development (i.e., digital features, PPD content, and education materials) that address the unique needs of vulnerable women through personal insights and social media interactions. The main findings of our study include themes of social support during pregnancy, management of the pregnancy journey through use of technology, and specific PPD topics of interest (e.g., symptomology, treatment), and unique opportunities to optimize digital and social media use for improving patient engagement and patient-provider relationships.

Our patient focus groups and interview themes are similar to ones reported in previous qualitative studies assessing information and technology needs for PPD management. In a UK study consisting of interviews with perinatal women and midwives, Doherty and colleagues found themes of “The pregnancy journey” (describing positive and negative experiences during the nine months of pregnancy, contrary to intuition that pregnancy is a purely positive experience), “Experience of Perinatal Care” (complex relationship with midwive professionals, lack of mental health screening in the clinical care setting), and “Technology Use in Pregnancy” (technology as provider of health information and communication with providers). These are analogous to our themes of “Use of Technology/Features”, “Pregnancy Education” and “Sources of Information: Doctor”. One major difference of this study is that providers interviewed were mostly midwives, reflecting the different structure of the UK national healthcare system. However, providers had very similar views on technology and PPD management as our interviewed providers. For example, midwives commented that many times they assessed the wellbeing of their patients based on their appearance (rather than performing a PPD screening) due to insufficient support services available. Therefore, technology could aid in improving this informal approach to mental health in the perinatal care setting.

### Differences and similarities between perspectives in different data sources

Our study analyzed data that ranged from the micro level of interviews to the macro level of social media. These datasets were deliberately selected to discover a wide range of information and technology needs for PPD management, coming from both patients and providers. We observed significant differences and similarities in participant themes across our data sources. Through our social media analysis, we observed that a general population of perinatal women used these channels to share their personal stories with PPD and obtain feedback regarding best choice of medical treatment, to vent about their family relationships (relationship with husband, in-laws), and in general to provide each other with social support. Our focus groups and interviews with vulnerable perinatal women, on the other hand, provided us with a more granular view of the information and technology needs of this specific population: better access to mental health resources including more streamlined communication with their providers, and PPD education materials that would alleviate their worries about their high-risk pregnancies. Some specific topics we saw repeatedly discussed in our social media dataset, which should be covered by PPD educational materials, information about psychiatric/psychological medications and their effects on breastfeeding, and the physical and mental health symptoms of PPD.

### Designing for young perinatal women

Our focus group and interview samples were comprised mostly of young women, indicating that our digital platform should be designed for the young adult female demographic. This population is accustomed to using digital tools to manage various aspects of their lives. The addition of a new tool for managing their mental health during the peripartum period would be a welcome addition to their available array, a finding that is in line with previous acceptability studies [55, 56]. Our participants also wanted products developed with evidence-based information and design. Many of the products available to them do not contain research-based behavioral change techniques, as we have found in our previous review of market PPD apps [35]. We expect that the specific behavior change techniques of feedback and monitoring, social support, information about health consequences, improving self-belief, comparison of behavior, and comparison of outcomes will be effective for our young target population and should therefore be incorporated as engagement elements in future digital health technologies for PPD management.

### Designing for interprofessional care delivery

As indicated by our individual provider interviews, digital technologies can offer providers a more streamlined and central care system. This is particularly necessary for the domain of maternal mental health, where various providers interact to coordinate care. This would improve diagnosing and treating PPD, such as having digital reminders to provide PPD screening and faster referrals to mental health professionals. They could also receive clinical decision support if symptomatic surveillance is conducted through a digital platform. For example, if screenings consistently indicated elevated depression symptoms, the provider could receive an alert to contact the patient. Such insights were concurred by patients, who reported experiencing difficulties with information silos in their care setting (ultrasounds shared via SMS; lab results shared via phone calls/regular mail). Because different caregivers monitor them at each appointment, it was difficult for them to interact and seek information from their caregiver team. Additionally, participants expressed that mental health was an area of care that was particularly not covered enough by their providers, in terms of patient education. Due to these shortcomings, a bi-directional communication channel between providers and patients would be a welcome digital feature.

### Designing for personalized support

Our participants reported having no digital tools which provided personalized support for PPD management. This was important to them, as each had had vastly different experiences during their pregnancies. For example, some had more support from family and friends than others, and for some it was their first pregnancy while others had had numerous pregnancies. They expressed a desire to receive daily motivational support, tailored to their health history and emotional needs. They mentioned they never receive such type of care through currently available clinical or commercial digital technologies. Therefore, we recommend that future digital platforms for PPD management would have to be designed to consider each individual’s medical history and social characteristics (i.e., previous experiences with PPD, previous pregnancies, income, insurance status).

Participants also wanted more clinical information about their pregnancies for reassurance and to cope with experiences such as miscarriages. All participants could relate to the theme of anxiety from prior miscarriages. Specifically, they desired to reduce anxiety if they had experienced a recent miscarriage and be able to be happy again if they became pregnant soon after a miscarriage. Additionally, they wanted more tools that would offer support in the postpartum period. This indicates that participants experience the pregnancy and postpartum period differently and desire that their support tools adapt to such differences.

### Limitations and future work

Our focus group and interviews were only done at one clinical site; therefore, more interviews should be conducted in different types of sites to better capture women’s views regarding PPD technologies. For example, a focus group can be conducted in a public health clinic for women. This would provide us with additional data to compare views of women attending an academic vs. non-academic practice. Another limitation is that our focus groups and interviews excluded Spanish-speaking participants and did not actively recruit other vulnerable groups such as immigrant participants. This limitation is applicable to both the patient and provider samples of this study. In future work, we aim to remediate this limitation by taking measures such as including Hispanic and immigrant women in our samples to ensure appropriate representation, and to address factors such as language barriers. An additional limitation of our study is a low sample size in our social media dataset, and possible representation bias as the content in our data may not be attributed to our target population of low-SES women. It is plausible that the remaining dataset contains additional themes that were not captured during the analysis. The rapid growth of digital technologies further complicates this issue, as the abundance of messages transmitted over the web and mobile media creates a data deluge, which may not be captured by the limited amount of publicly available interactions we harvested and coded. Consequently, for large datasets, it becomes necessary to complement qualitative methods with automated machine learning techniques to obtain generalizable insights [41, 57]. Also, we are not able to construe posters’ sociodemographic characteristics from our social media data, however, our selection of social media channels is informed by focus group and interview insights. As indicated by recent research [58], it is important to consider omnichannel strategies to increase reach and impact of interventions and improve diversity, equity, and inclusion of vulnerable populations affected by health disparities. The next steps in our research program will be to leverage our findings for optimal design paradigms in the prevention, self-management, and care coordination for PPD. Linking our inductively derived themes from our multimodal data analysis to theory-linked strategies and social determinants of health is vital so we can enable intervention development and implementation, with an ultimate goal to address disparities and improve health outcomes among women at risk for PPD.

## Conclusion

Our study offers a novel compilation of stakeholder perspectives that can assist with advancing PPD management through tools that are accessible to vulnerable populations, that offer credible PPD education, social support, and that can alleviate the current strain in healthcare resources that clinical providers face when managing PPD. Our resulting themes of sharing stories and narratives and use of technology highlight a current landscape of maternal mental health management where women desire to feel connected to other groups of peripartum women. Our study suggests that women need different opportunities and settings to share and acquire information on their pregnancy and mental health. For example, the anonymous nature of social media may make some feel more comfortable in making the first step of disclosing their symptoms. At the same time, a smaller and in-person support group of peers may be an ideal setting to share *local* resources. Such findings may also be applicable to female patients outside the peripartum domain. This represents an opportunity for the promotion of positive health behaviors and evidence-based practices that can prevent or reduce public health problems such as PPD. By their ability to remove barriers of time and space, digital technologies play a role as facilitators of key prevention and monitoring processes, including education, self-monitoring, and social support.

## Electronic supplementary material

Below is the link to the electronic supplementary material.

Supplementary Material 1: Appendix A

Supplementary Material 2: Appendix B

Supplementary Material 3: Appendix C

## Data Availability

The datasets generated and/or analyzed during the current study are not publicly available to help protect individual privacy, but are available from the corresponding author on reasonable request.
