# Supplementary material for: Digital health technologies for peripartum depression management among low-socioeconomic populations: perspectives from patients, providers, and social media channels
Source: BMC Pregnancy Childbirth. 2023 Jun 3;23:411. doi: 10.1186/s12884-023-05729-9 (PMC10239590; doi:10.1186/s12884-023-05729-9)
Supplement: Supplementary file 1 — Supplementary Material 1: Appendix A [file 12884_2023_5729_MOESM1_ESM.docx]

Appendix A- Patient Survey on Demographics, Pregnancy, and Experiences with Peripartum Depression

Start of Block: Default Question Block

Q1 What is your age?

- 18-24 years old (1)
- 25-34 years old (2)
- 35-44 years old (3)
- 45+ years old (4)

Q3 What is your gender?

- Male (1)
- Female (2)
- Other (3) ________________________________________________
- Prefer not to answer (4)

Q4 Which best describes your race?

- White (1)
- Black or African American (2)
- American Indian or Alaska Native (3)
- Asian (4)
- Native Hawaiian or Pacific Islander (5)
- Other (6) ________________________________________________
- Prefer not to answer (7)

Q5 Are you Hispanic or Latino?

- Yes (1)
- No (2)

Q22 Education level (please select the highest you have completed)

- No schooling completed (1)
- Nursery school to 8th grade (2)
- Some high school, no diploma (3)
- High School Graduate, diploma or the equivalent (Example: GED) (4)
- Some college credit, no degree (5)
- Trade/Technical/Vocational training (6)
- Associate Degree (7)
- Bachelor's Degree (8)
- Master's Degree (9)
- Professional Degree (10)
- Doctoral Degree (11)

Q23 Employment Status

- Employed for wages (1)
- Self-employed (2)
- Out of work and looking for work (3)
- Out of work but not currently looking for work (4)
- Homemaker (5)
- Student (6)
- Military (7)
- Retired (8)
- Unable to work (9)
- Prefer not to answer (10)

Q6 What is your marital status?

- Single (Never Married) (1)
- Married (2)
- Widowed (3)
- Divorced (4)
- Separated (5)

Q24 Number of people currently living in your home:

________________________________________________________________

Q2 What is your annual household income?

- Under $20,000 (1)
- $20,001-$40,000 (2)
- $40,001-$60,000 (3)
- $60,001-$80,000 (4)
- Over $80,000 (5)

Q25 What languages do you speak in your home?

________________________________________________________________

Q26 What is your current Zip code?

________________________________________________________________

Q7 How many children do you have?

________________________________________________________________

Q8 How many pregnancies have you had?

________________________________________________________________

Q9 Are you currently pregnant?

- Yes (1)
- No (2)

Q10 How long ago did you have your most recent child?

________________________________________________________________

Q11 With your most recent/current pregnancy, have you experienced episodes of depression, feeling down, or what is sometimes called the baby blues?

- Yes (1)
- No (2)

Display This Question:

If With your most recent/current pregnancy, have you experienced episodes of depression, feeling dow... = Yes

Q13 When did you experience these episodes?

- During pregnancy (1)
- After delivery (2)
- Both (3)

Display This Question:

If With your most recent/current pregnancy, have you experienced episodes of depression, feeling dow... = Yes

Q16 Did you speak with a doctor about these feelings?

- Yes (1)
- No (2)

Q17 Did you have episodes of depression or feeling down with your previous pregnancies or following the birth of other children?

- Yes (1)
- No (2)

Q18 When you are interested in learning more about something like your pregnancy or how you are feeling do you:

- Look for information on the Internet (1)
- Speak with your Doctor (2)
- Look in books or other written source of information (3)
- Speak with friends or family (4)
- Look for information from social media (5)

Q19 Do you currently own a cell phone?

- Yes (1)
- No (2)

Q20 Do you use apps like:

- Games (1)
- Kindle (2)
- Spotify (3)
- Pregnancy related apps (4)
- Healthcare apps (tracking activity) (5)
- Banking apps (6)
- Uber/Lyft (7)
- Shopping apps (Amazon, Target, Walmart) (8)
- Netflix (9)
- Google Maps (10)
- Snapchat (11)
- Instagram (12)
- Facebook (13)
- Twitter (14)
- WhatsApp (15)

Q21 What is your favorite app?

________________________________________________________________

End of Block: Default Question Block
