# Supplementary material for: Digital health technologies for peripartum depression management among low-socioeconomic populations: perspectives from patients, providers, and social media channels
Source: BMC Pregnancy Childbirth. 2023 Jun 3;23:411. doi: 10.1186/s12884-023-05729-9 (PMC10239590; doi:10.1186/s12884-023-05729-9)
Supplement: Supplementary file 2 — Supplementary Material 2: Appendix B [file 12884_2023_5729_MOESM2_ESM.pptx]

## Slide 1
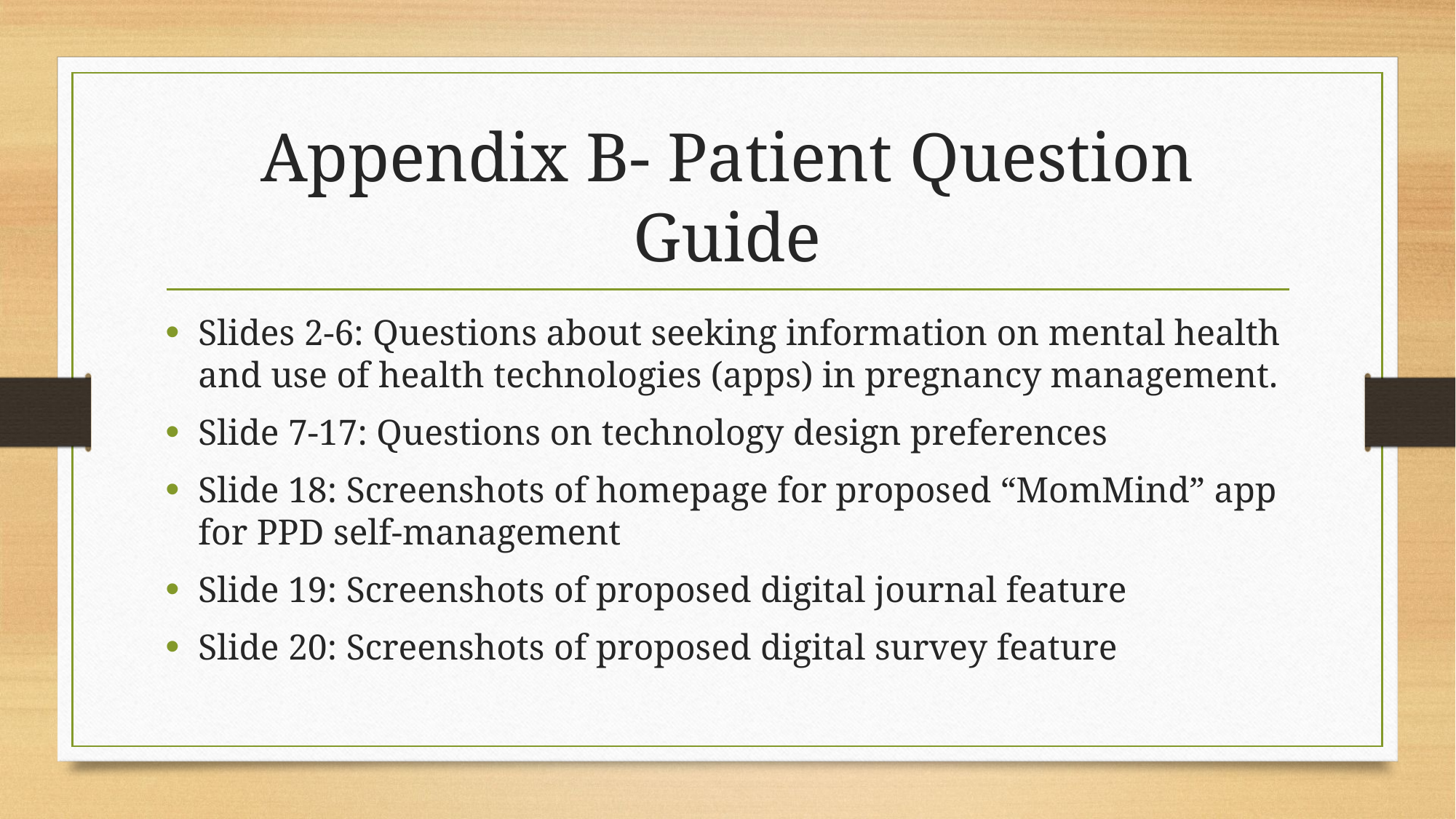

# Appendix B- Patient Question Guide
Slides 2-6: Questions about seeking information on mental health and use of health technologies (apps) in pregnancy management.
Slide 7-17: Questions on technology design preferences
Slide 18: Screenshots of homepage for proposed “MomMind” app for PPD self-management
Slide 19: Screenshots of proposed digital journal feature
Slide 20: Screenshots of proposed digital survey feature

## Slide 2
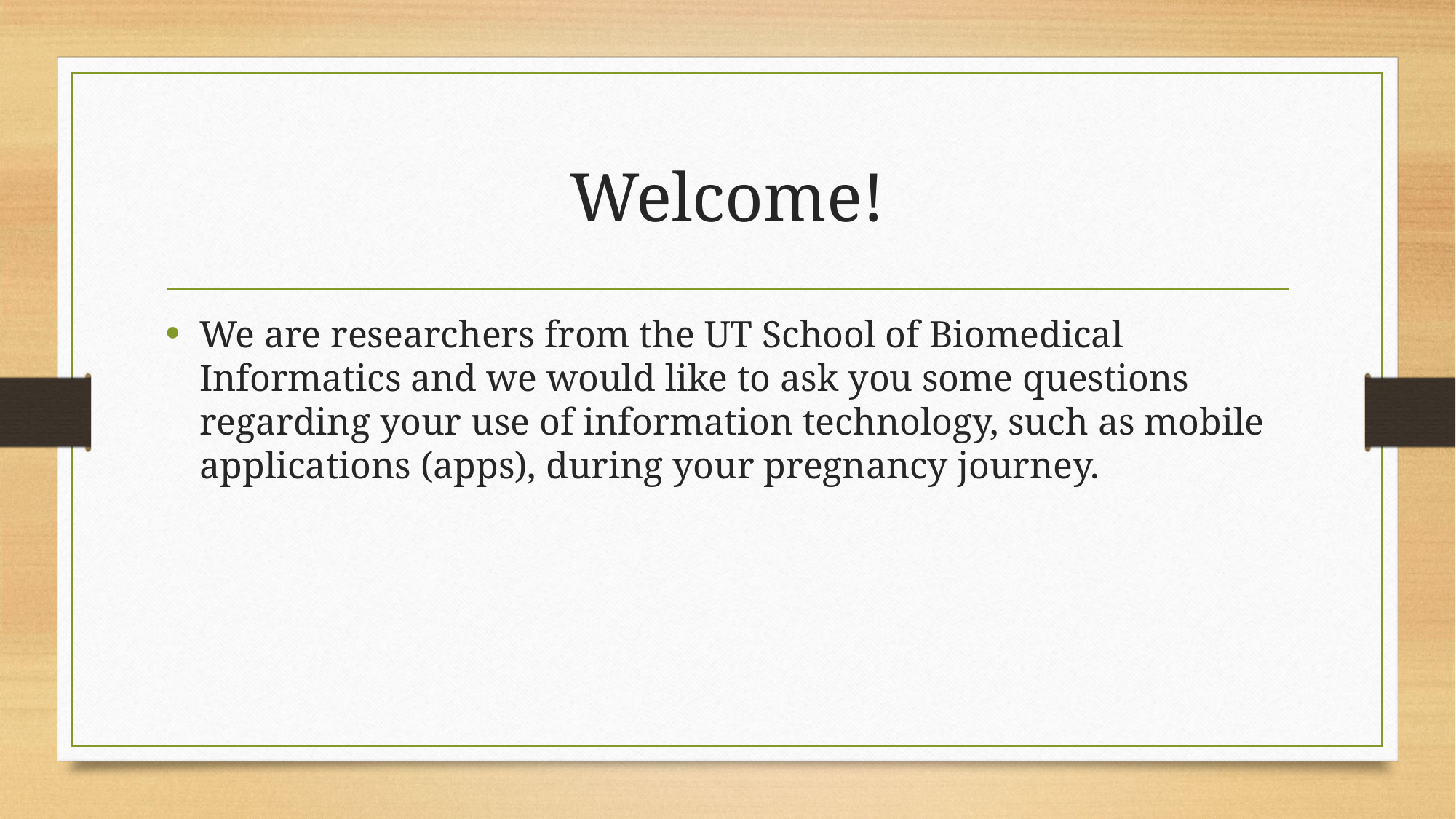

# Welcome!
We are researchers from the UT School of Biomedical Informatics and we would like to ask you some questions regarding your use of information technology, such as mobile applications (apps), during your pregnancy journey.

## Slide 3
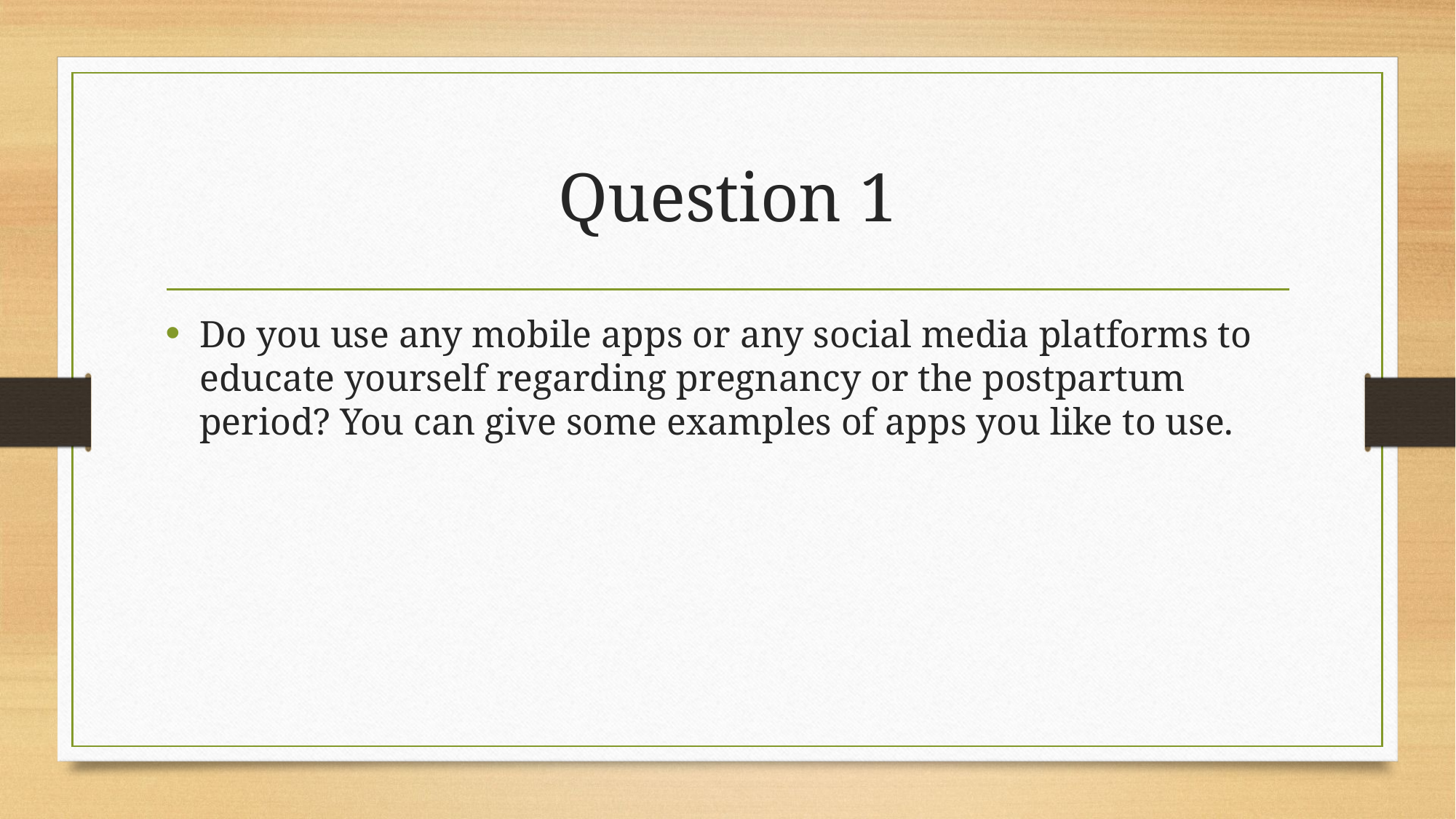

# Question 1
Do you use any mobile apps or any social media platforms to educate yourself regarding pregnancy or the postpartum period? You can give some examples of apps you like to use.

## Slide 4
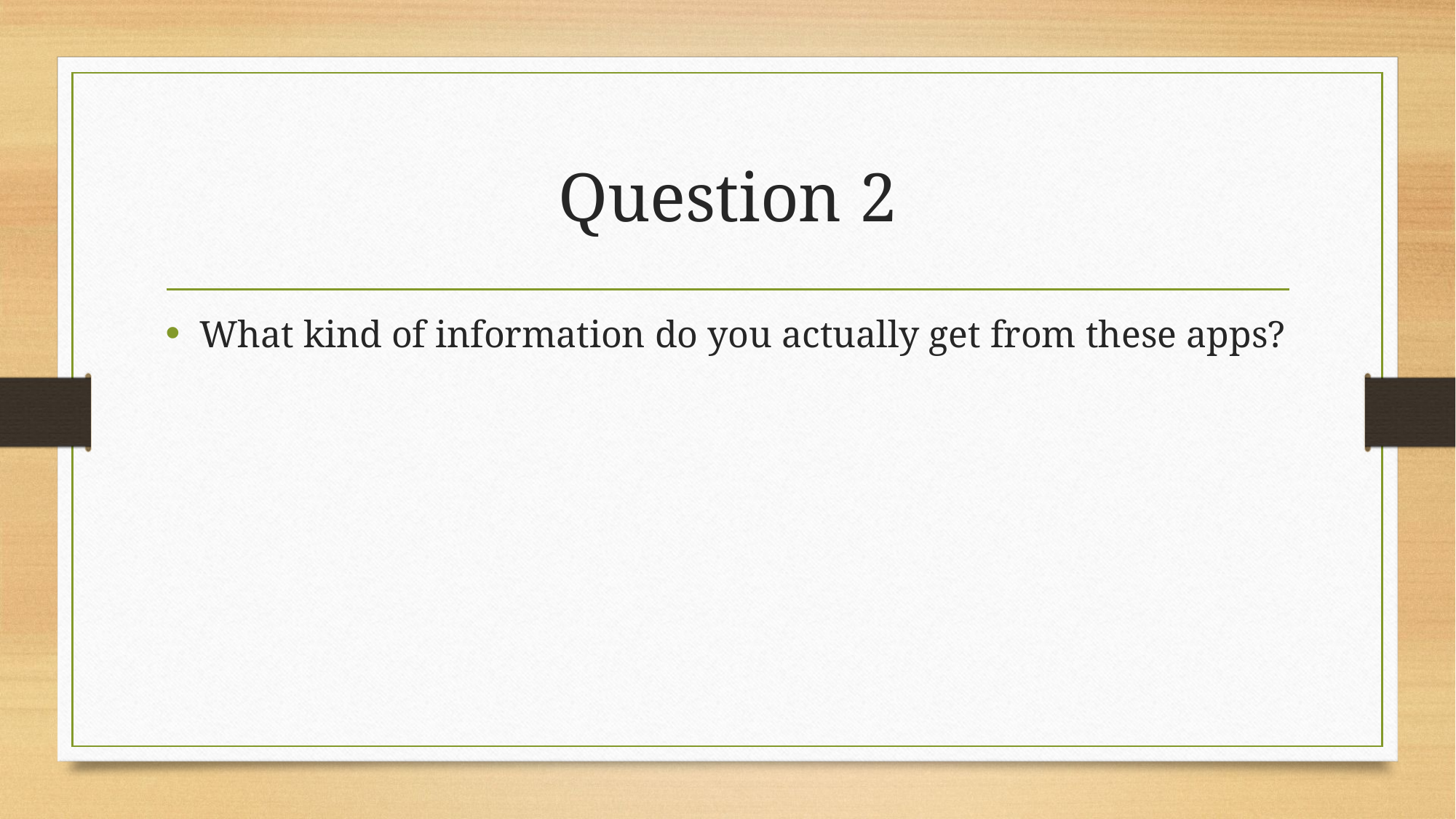

# Question 2
What kind of information do you actually get from these apps?

## Slide 5
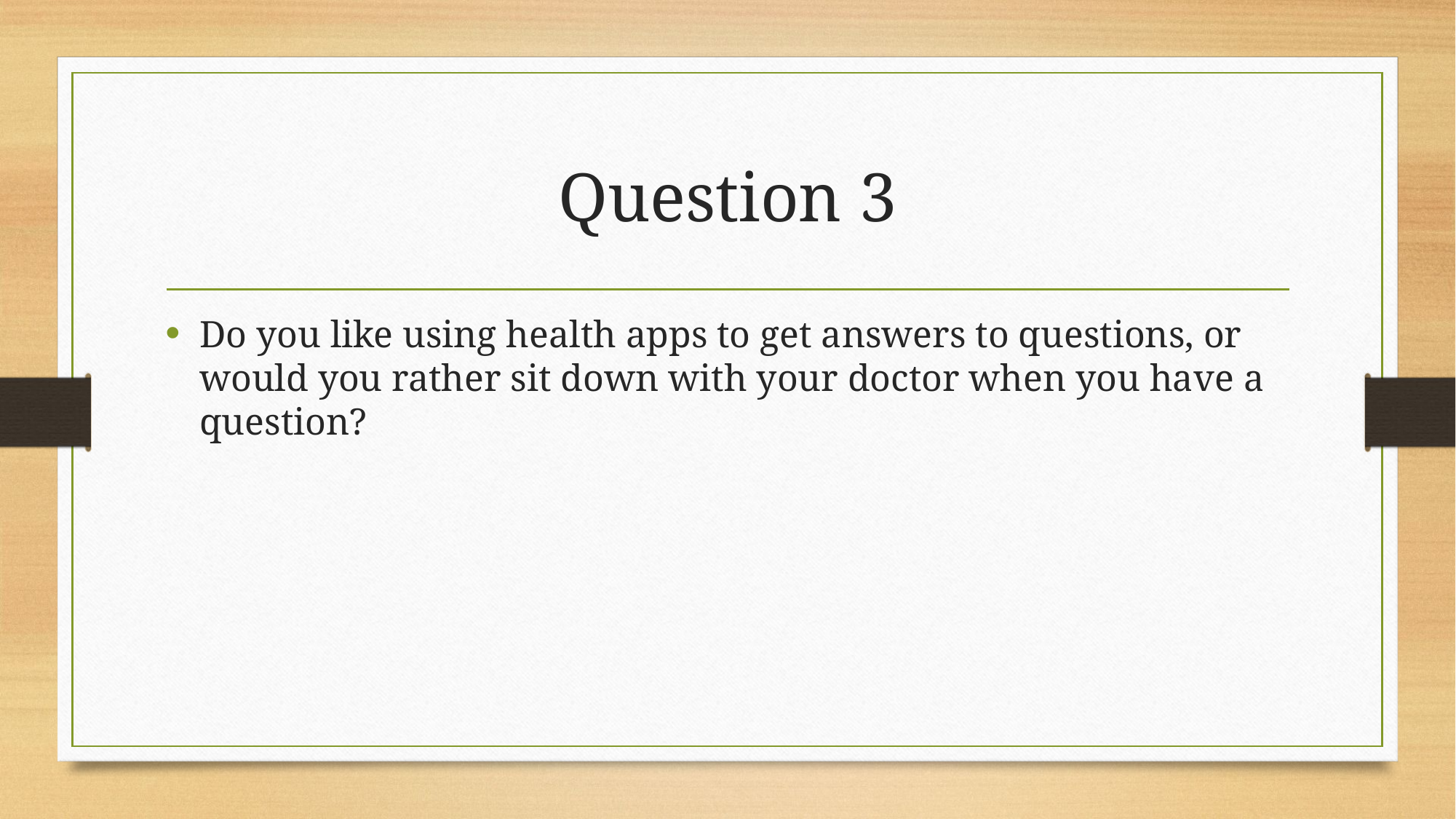

# Question 3
Do you like using health apps to get answers to questions, or would you rather sit down with your doctor when you have a question?

## Slide 6
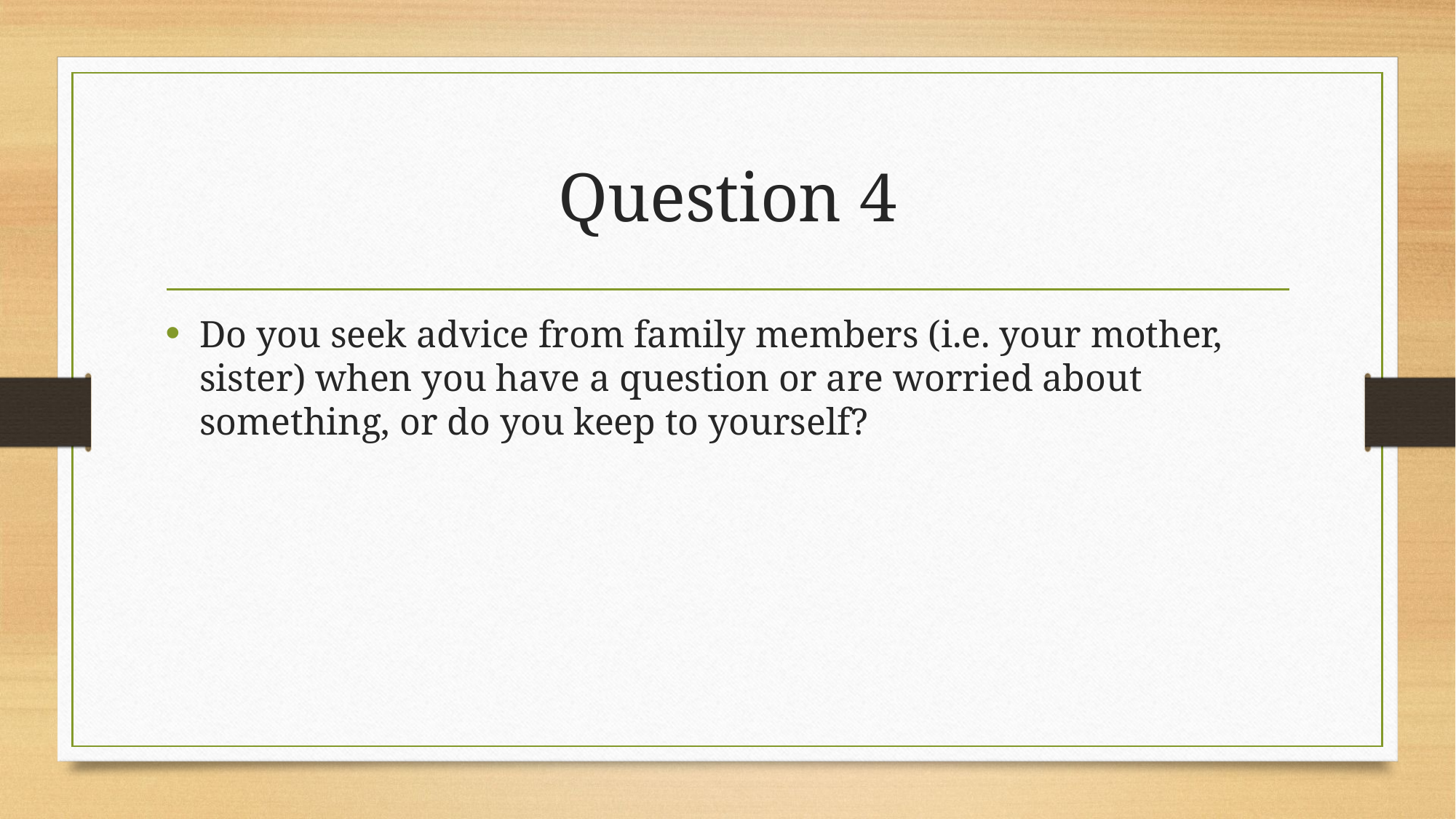

# Question 4
Do you seek advice from family members (i.e. your mother, sister) when you have a question or are worried about something, or do you keep to yourself?

## Slide 7
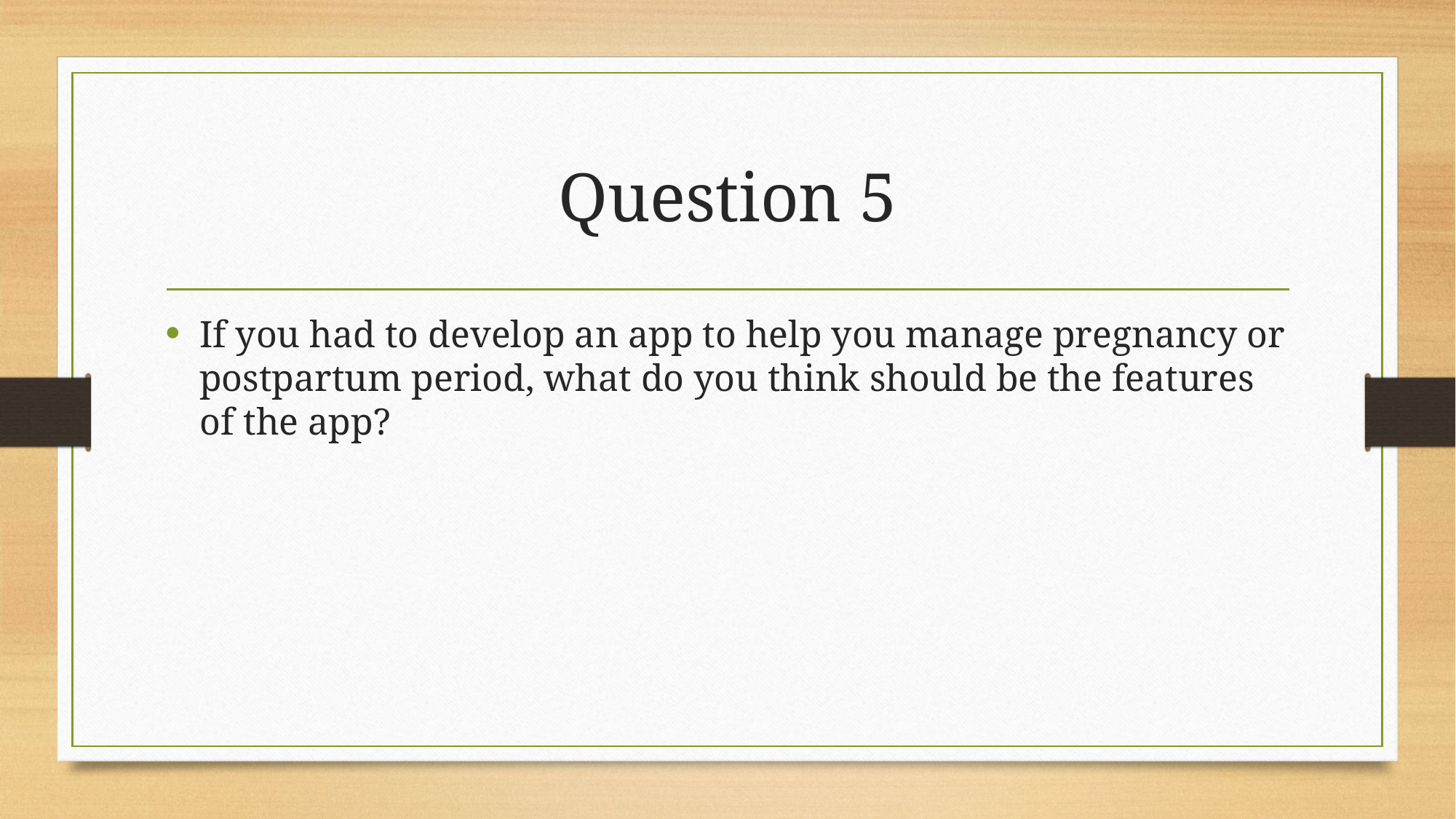

# Question 5
If you had to develop an app to help you manage pregnancy or postpartum period, what do you think should be the features of the app?

## Slide 8
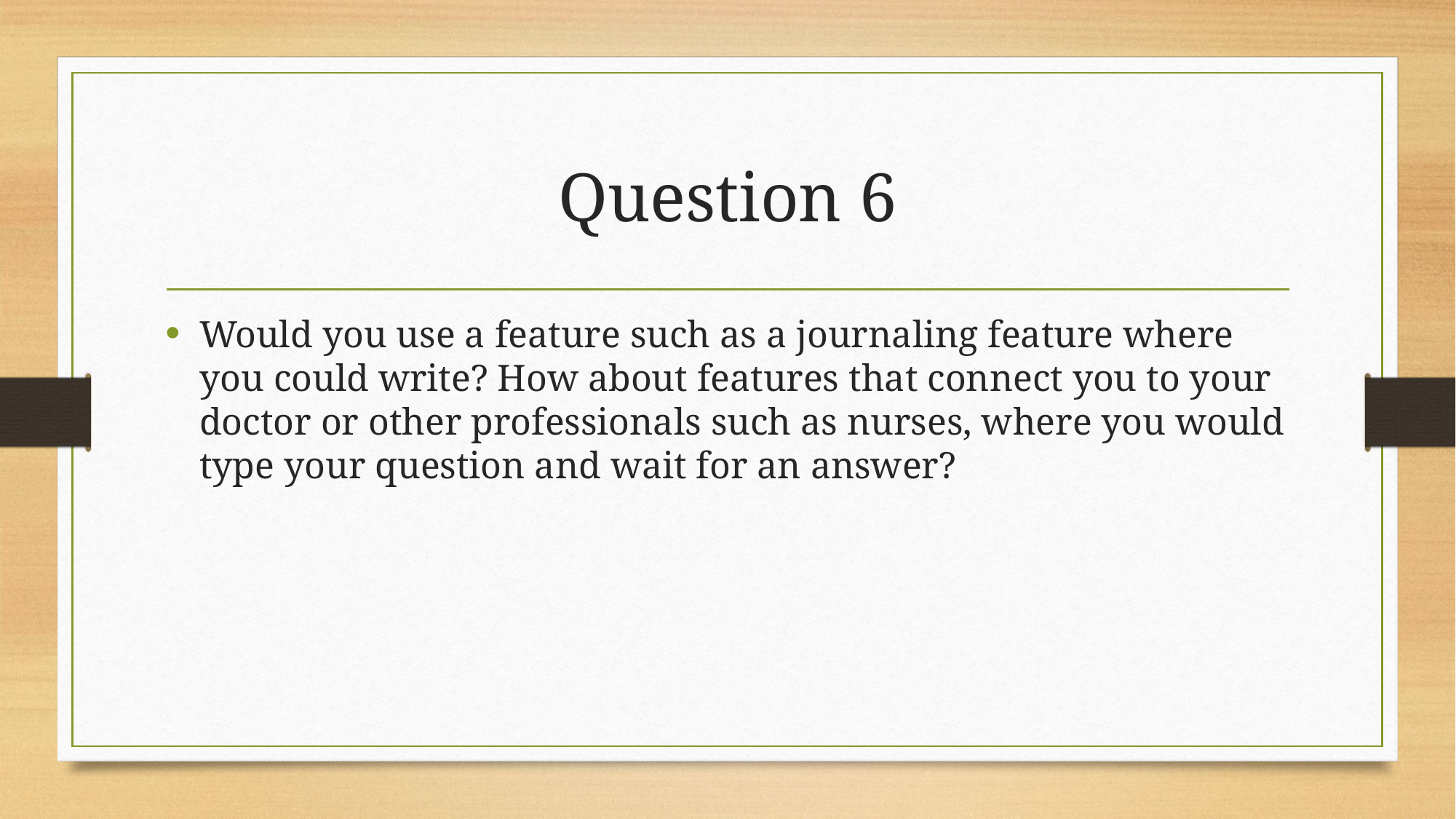

# Question 6
Would you use a feature such as a journaling feature where you could write? How about features that connect you to your doctor or other professionals such as nurses, where you would type your question and wait for an answer?

## Slide 9
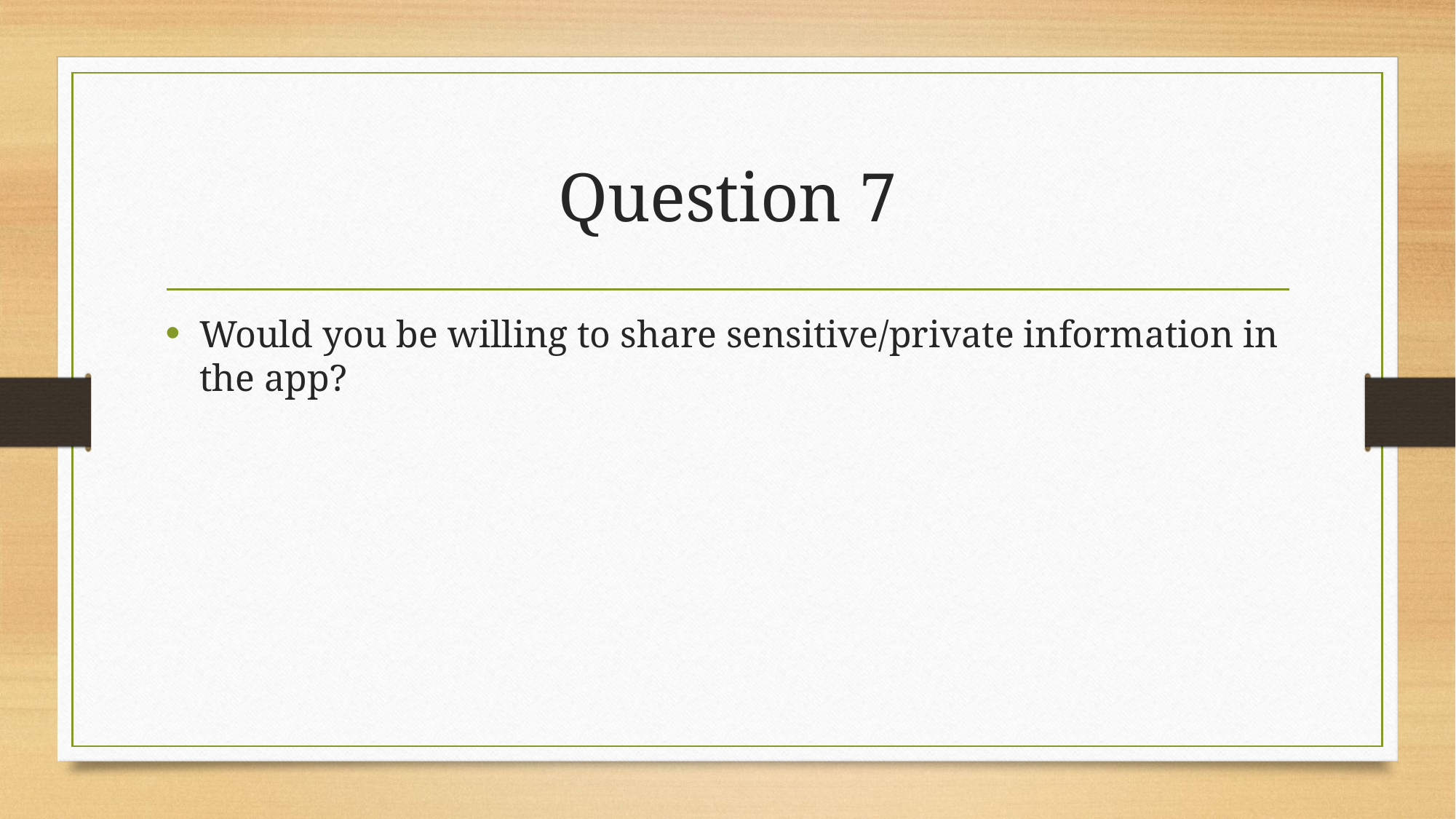

# Question 7
Would you be willing to share sensitive/private information in the app?

## Slide 10
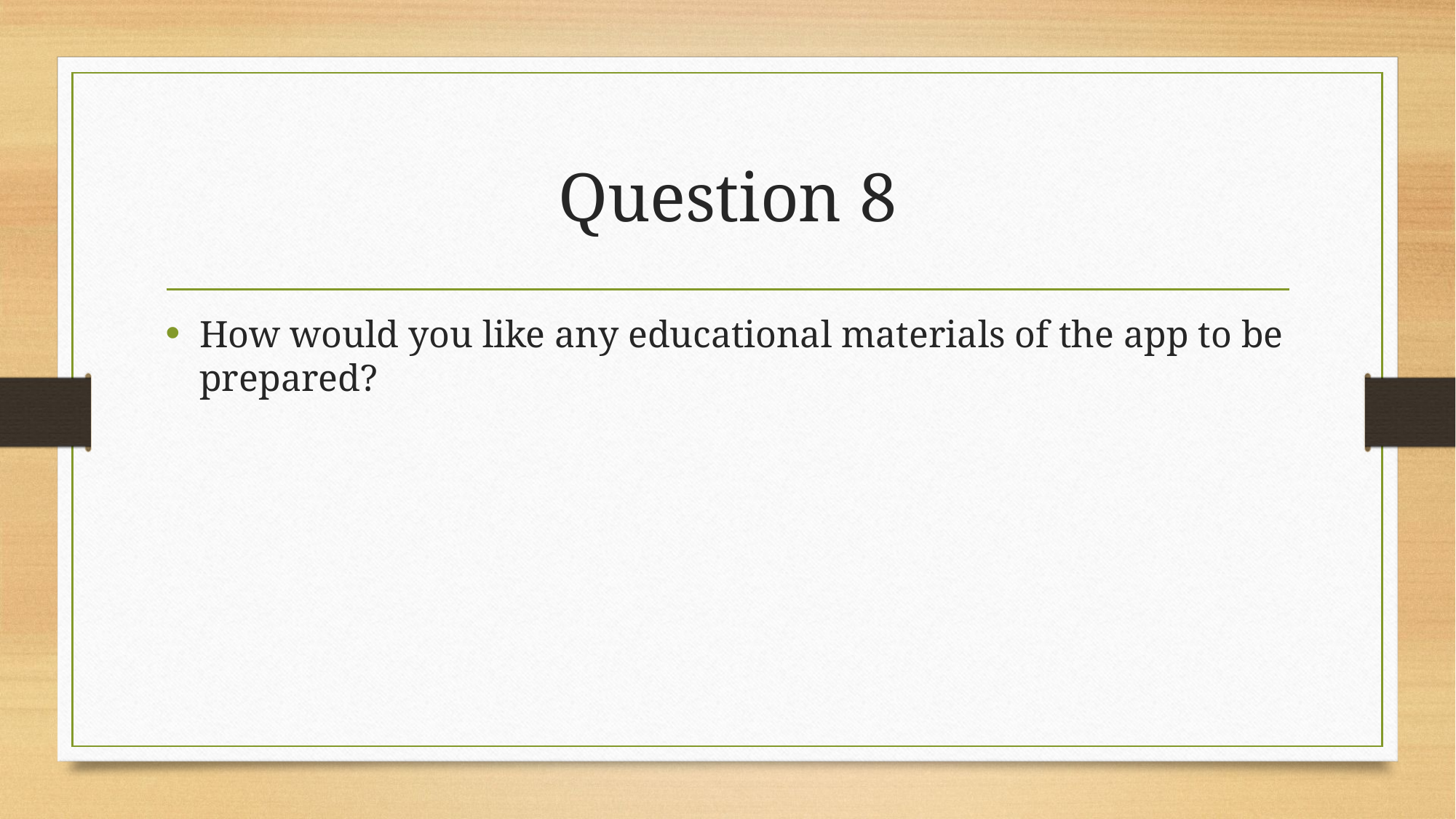

# Question 8
How would you like any educational materials of the app to be prepared?

## Slide 11
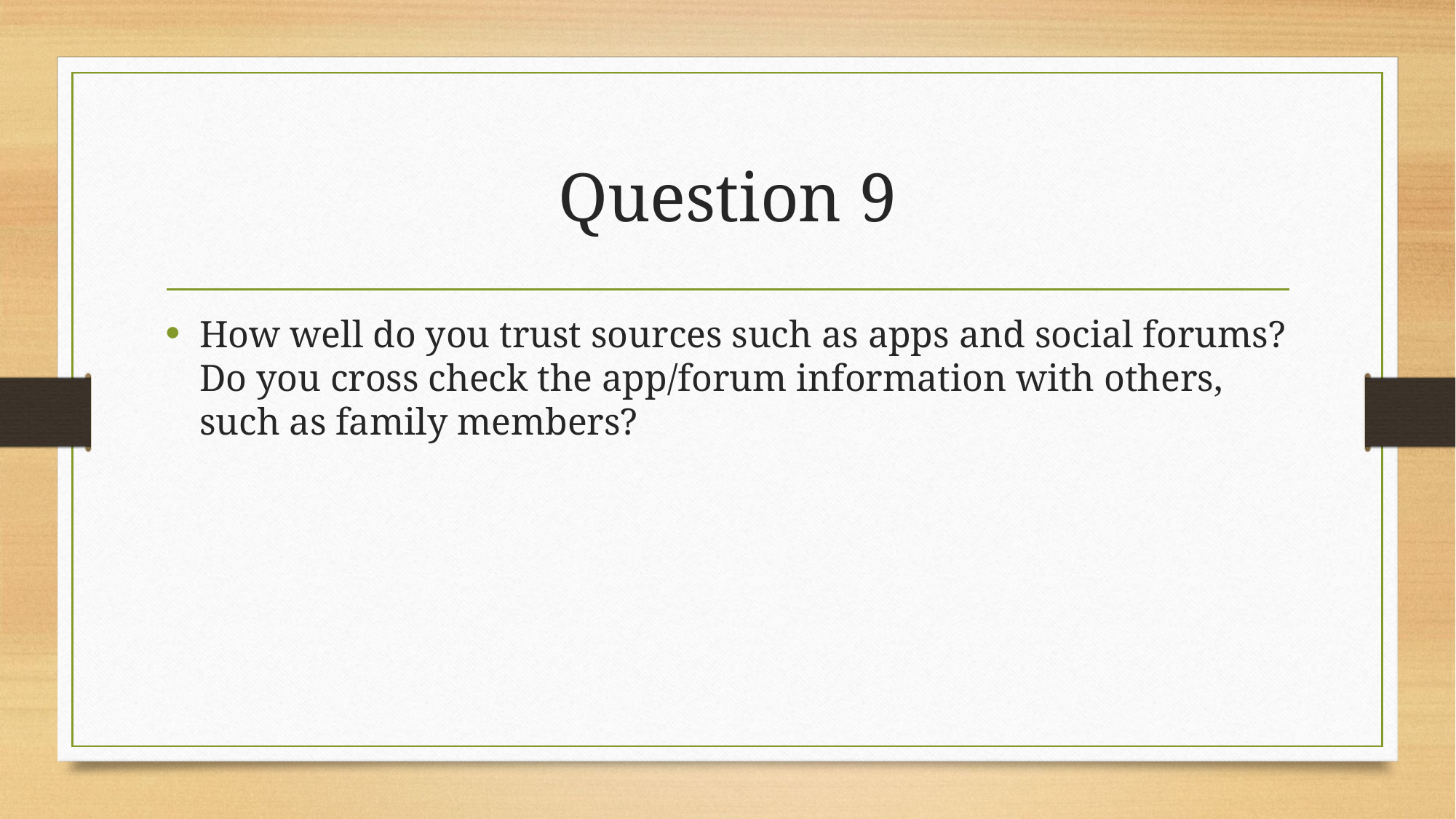

# Question 9
How well do you trust sources such as apps and social forums? Do you cross check the app/forum information with others, such as family members?

## Slide 12
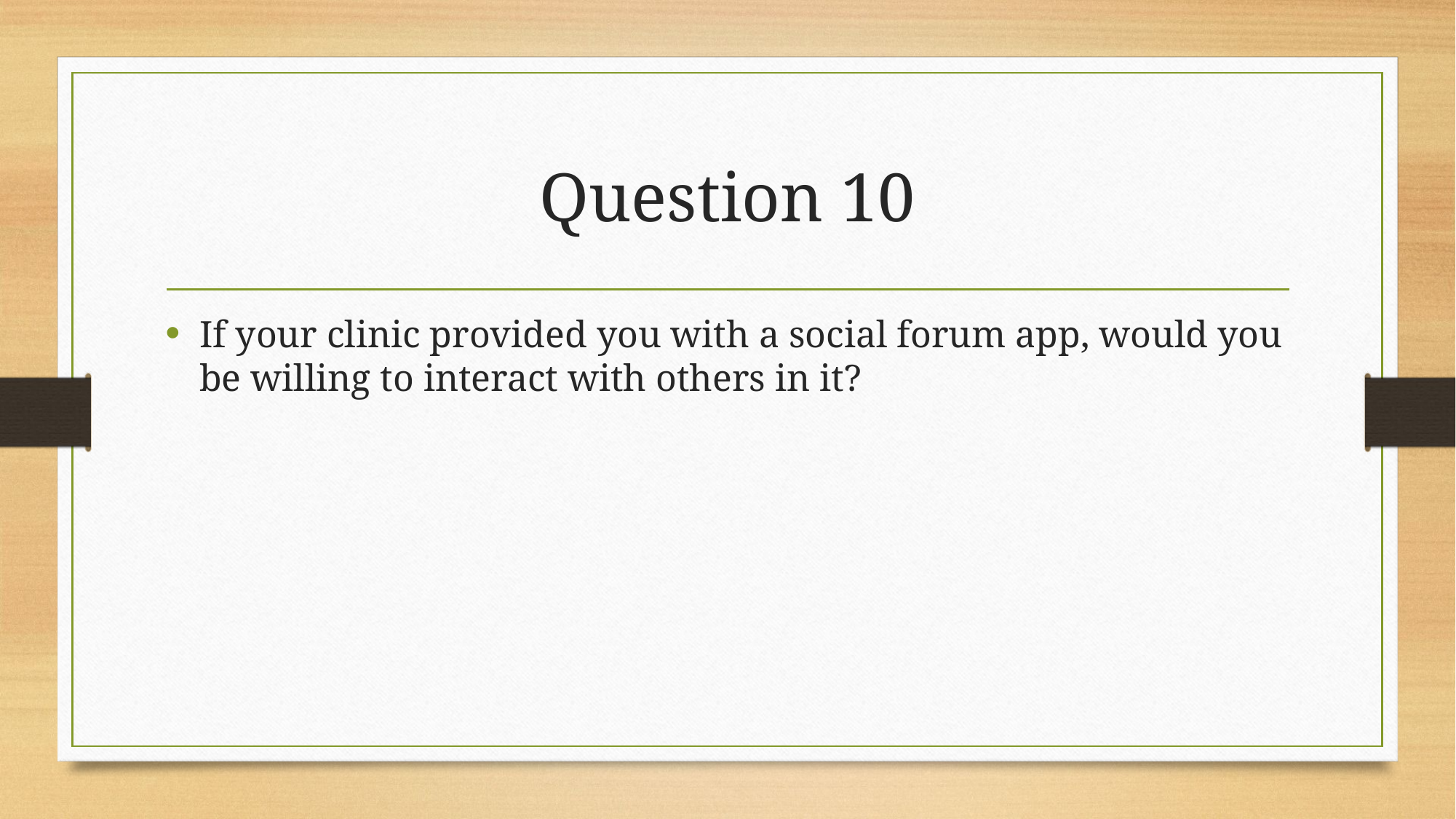

# Question 10
If your clinic provided you with a social forum app, would you be willing to interact with others in it?

## Slide 13
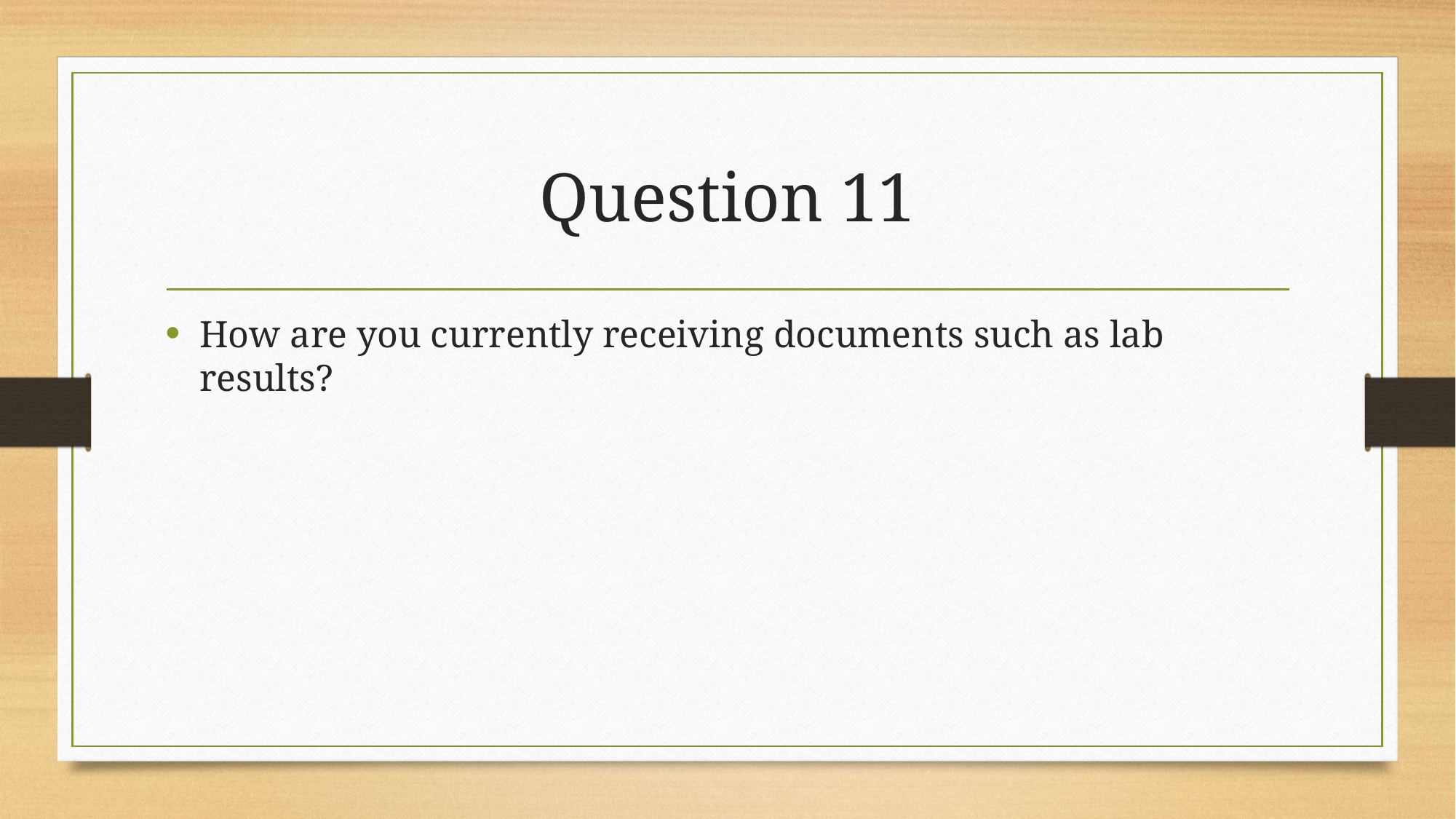

# Question 11
How are you currently receiving documents such as lab results?

## Slide 14
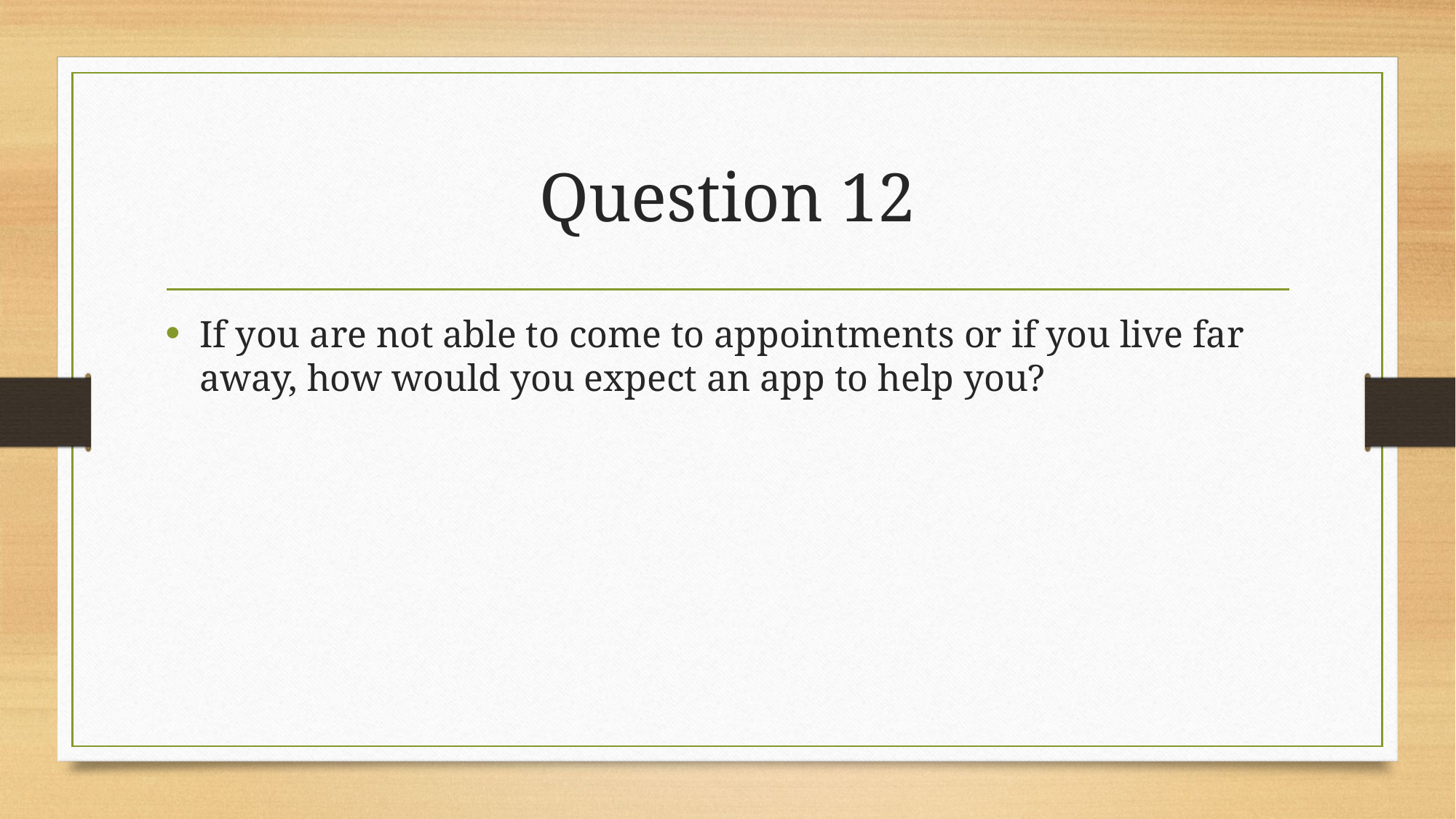

# Question 12
If you are not able to come to appointments or if you live far away, how would you expect an app to help you?

## Slide 15
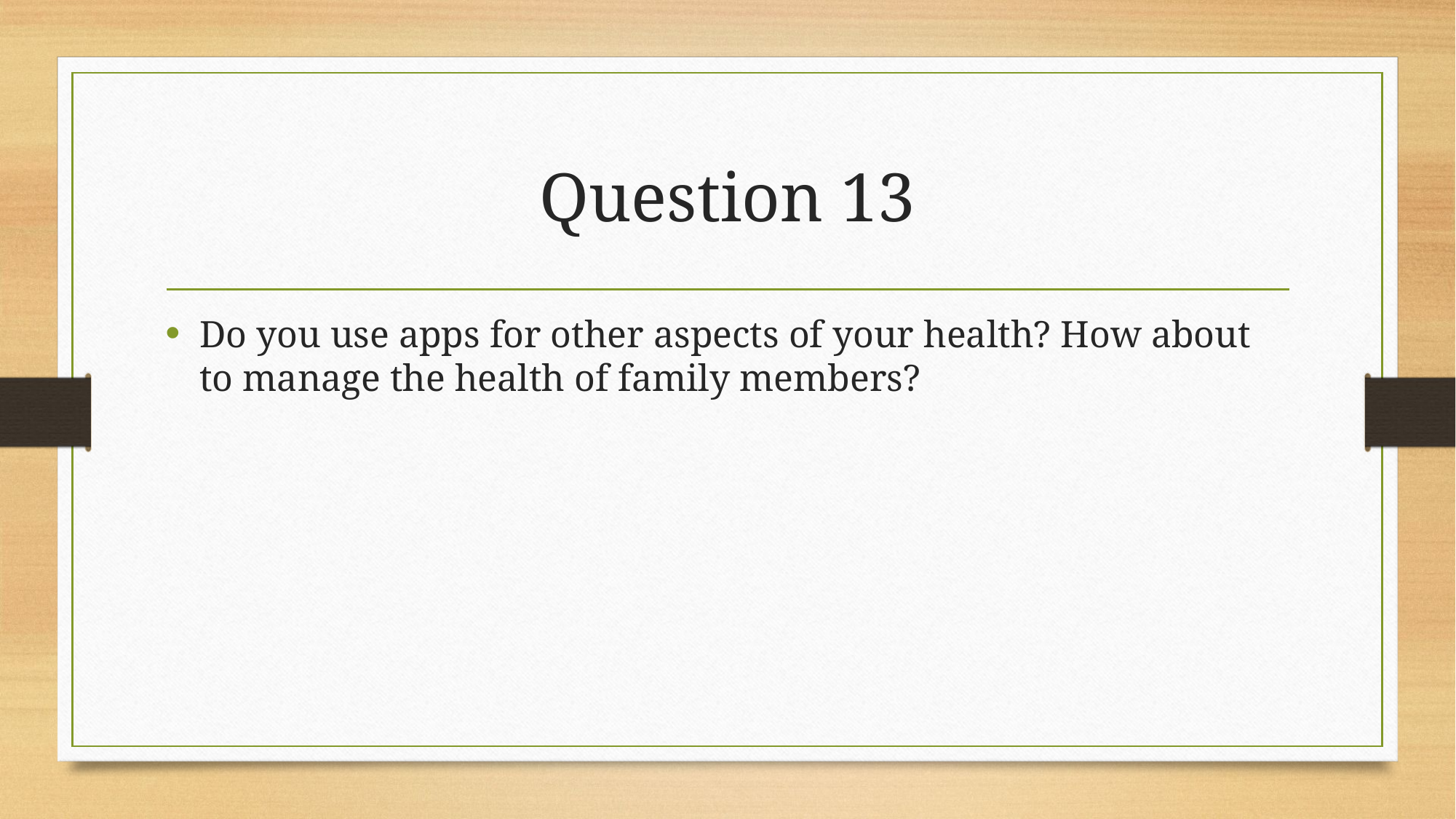

# Question 13
Do you use apps for other aspects of your health? How about to manage the health of family members?

## Slide 16
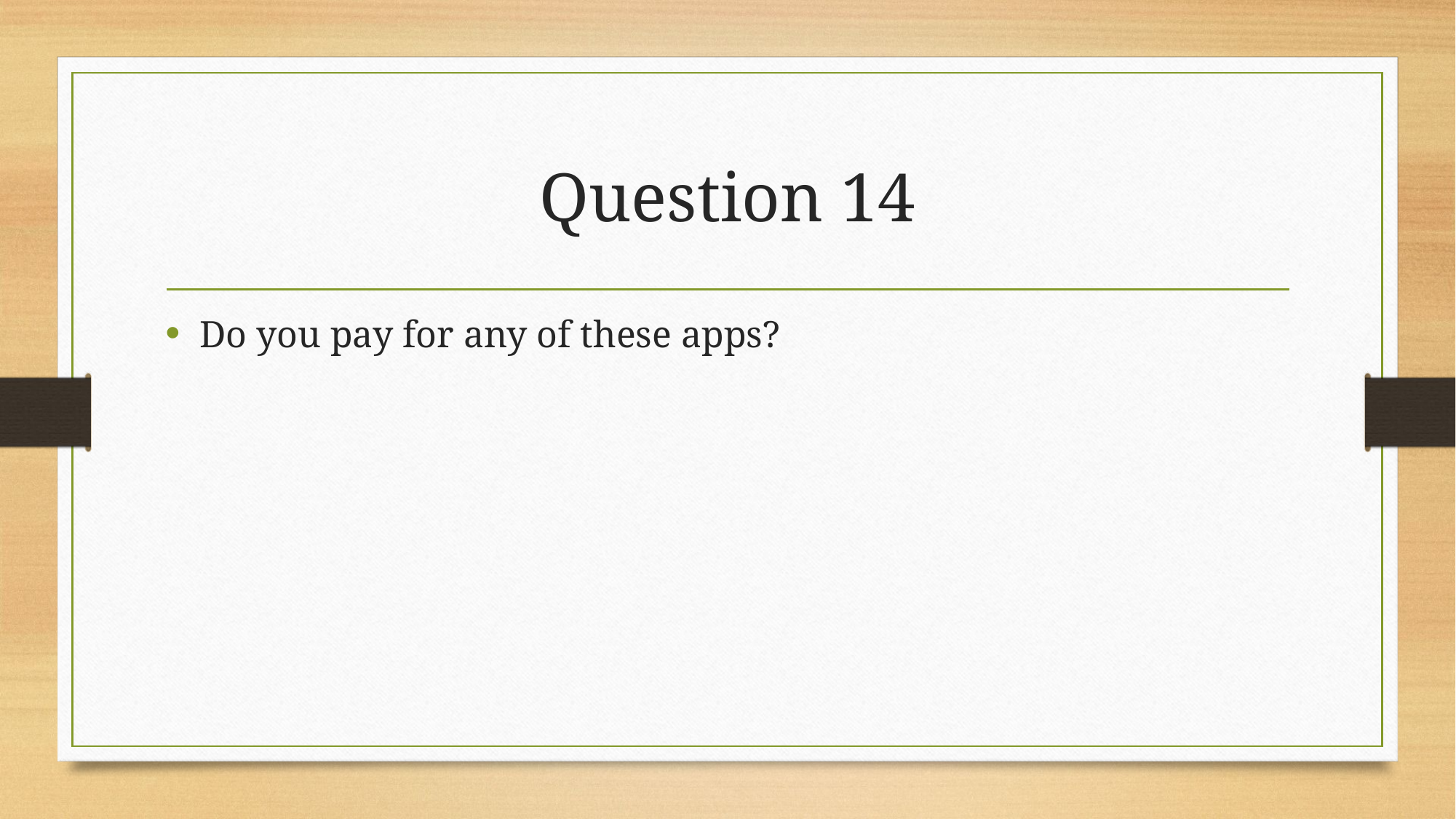

# Question 14
Do you pay for any of these apps?

## Slide 17
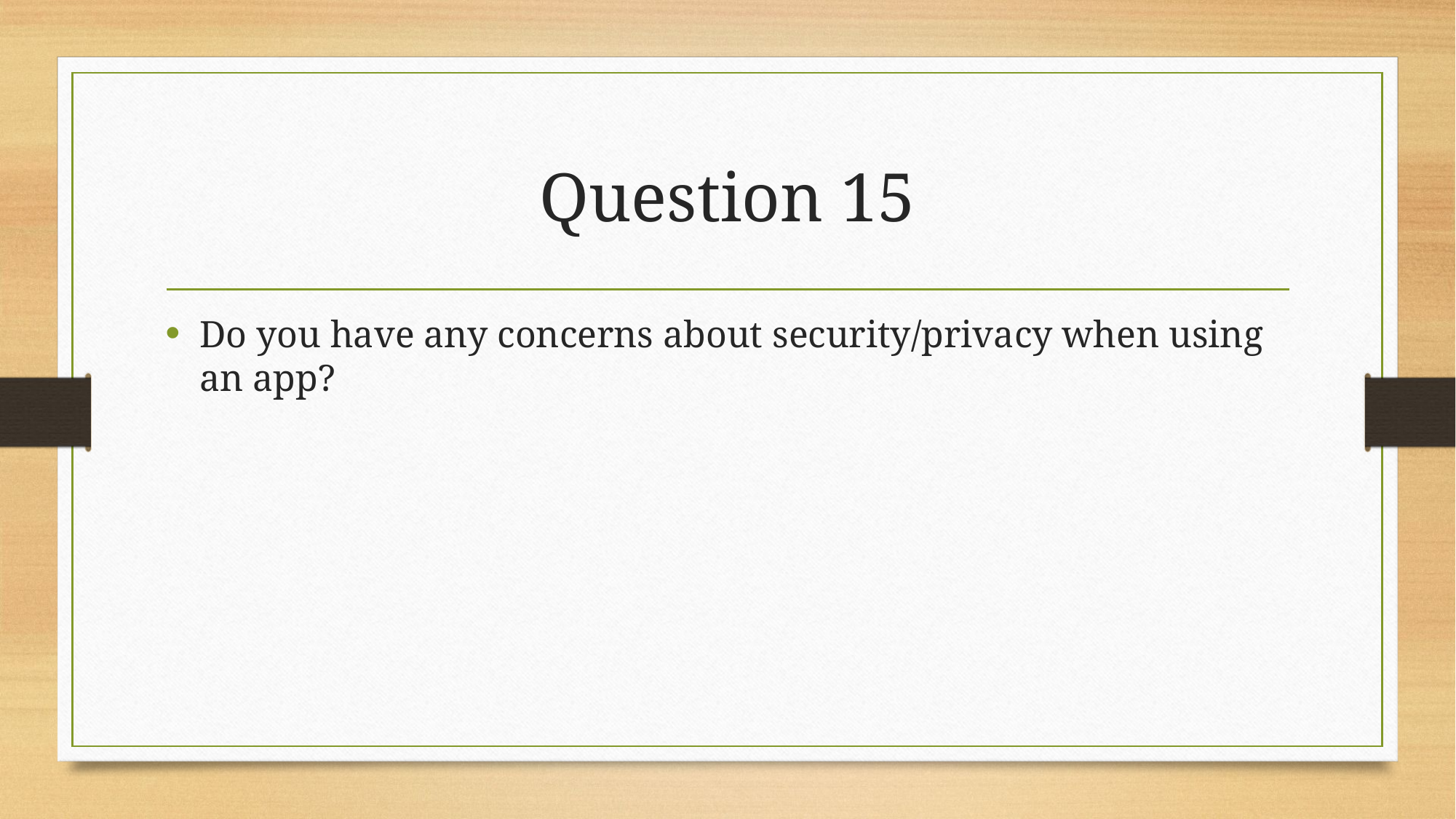

# Question 15
Do you have any concerns about security/privacy when using an app?

## Slide 18
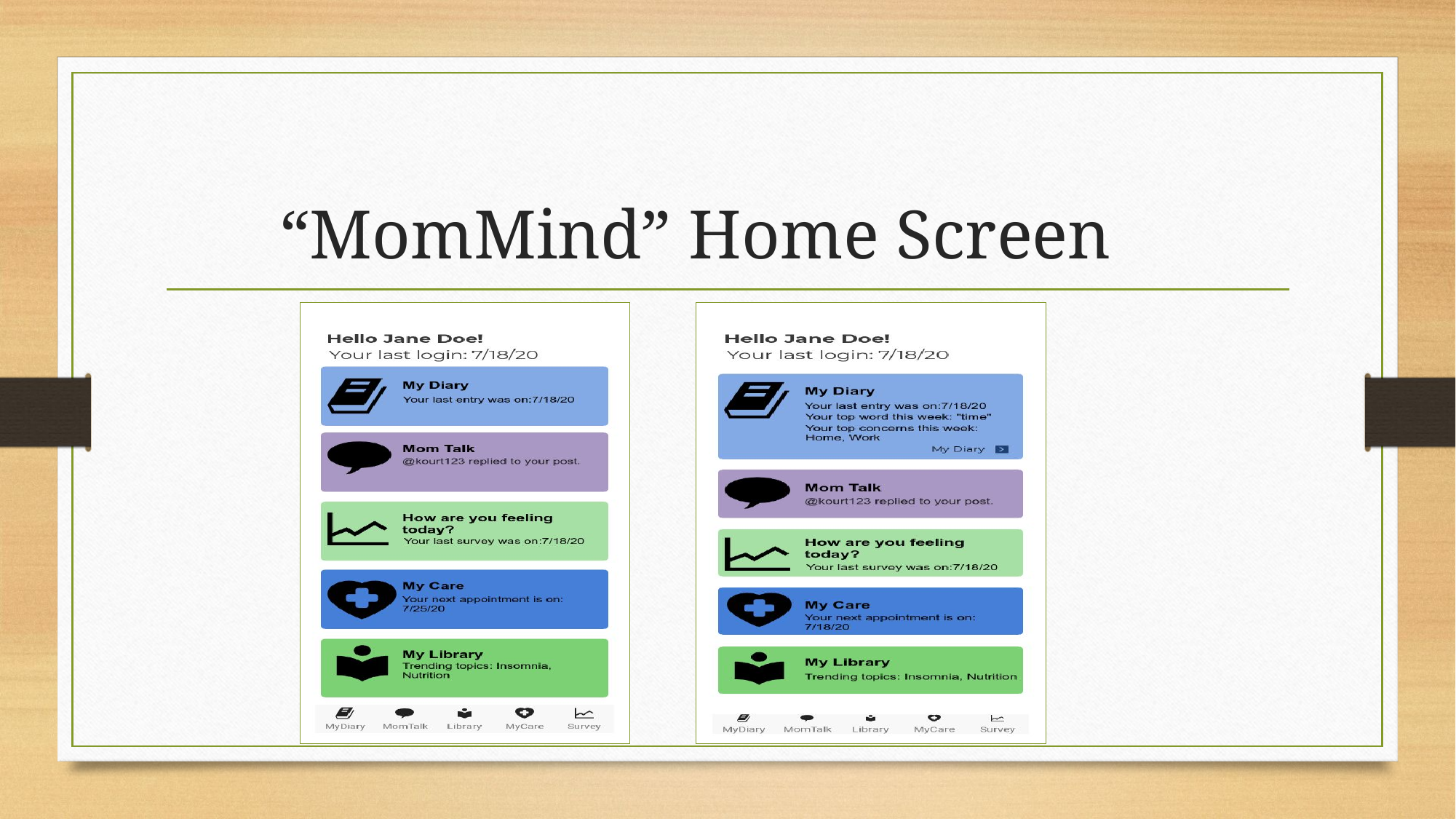

# “MomMind” Home Screen

## Slide 19
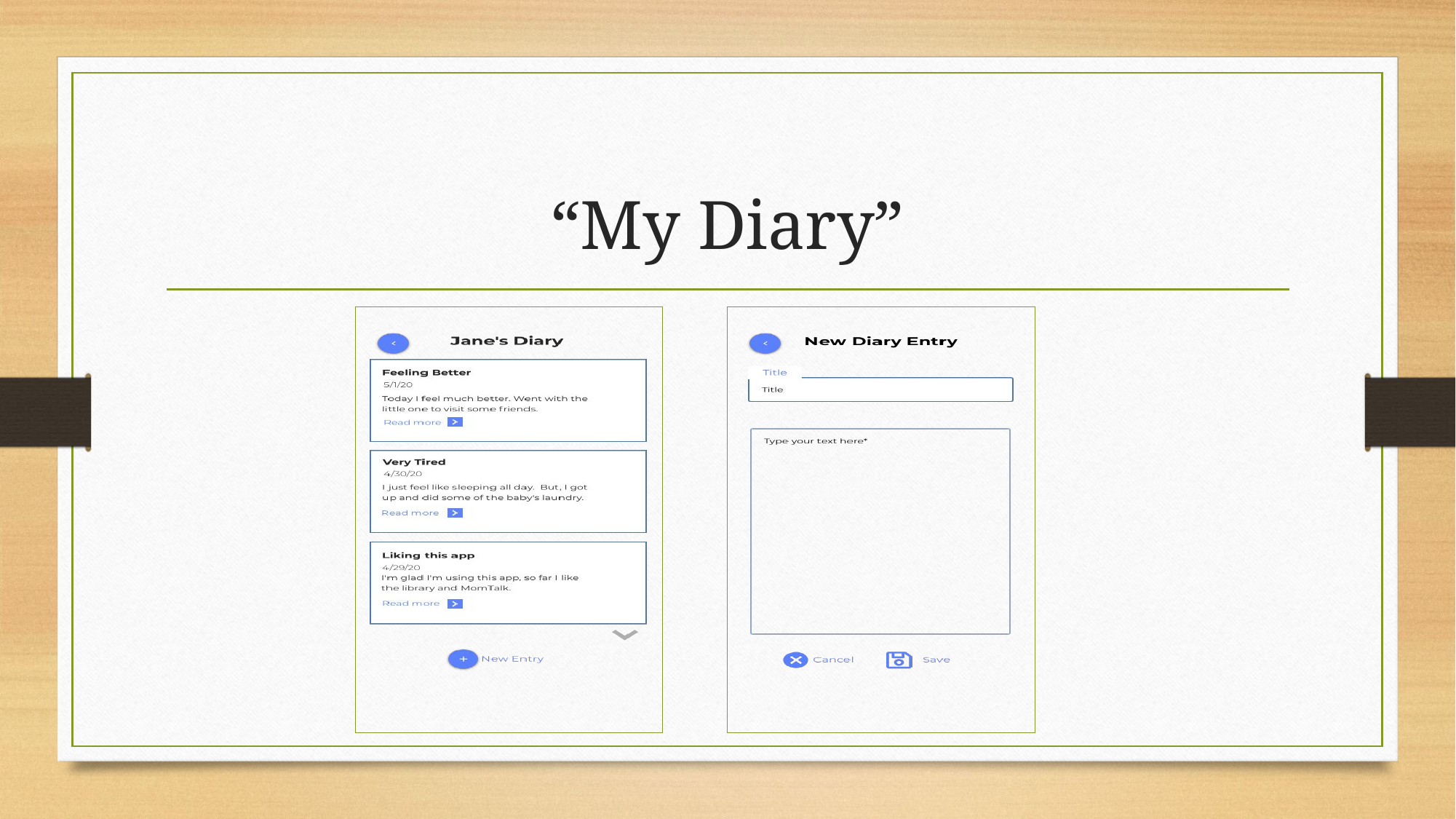

# “My Diary”

## Slide 20
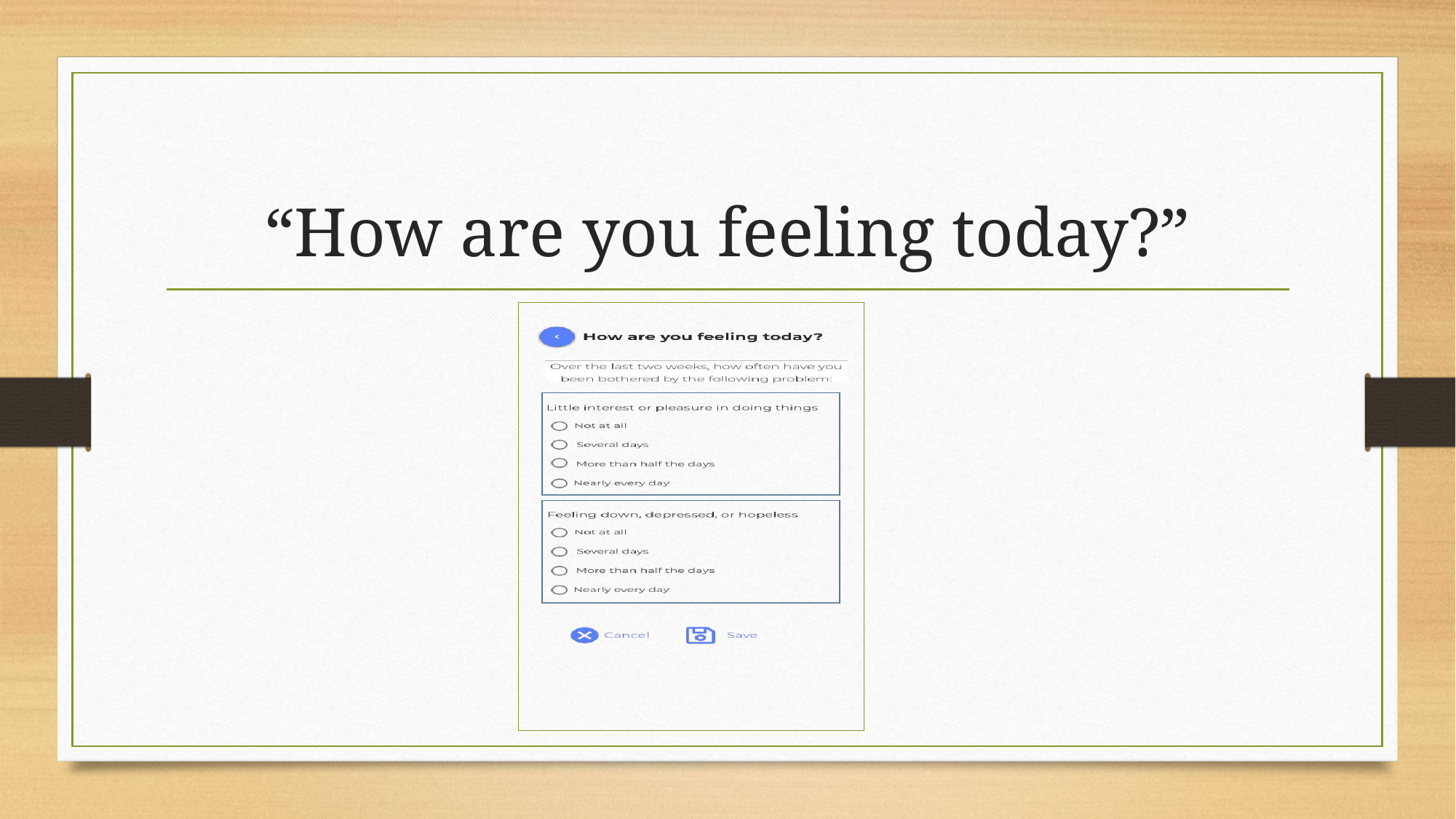

# “How are you feeling today?”

## Slide 21
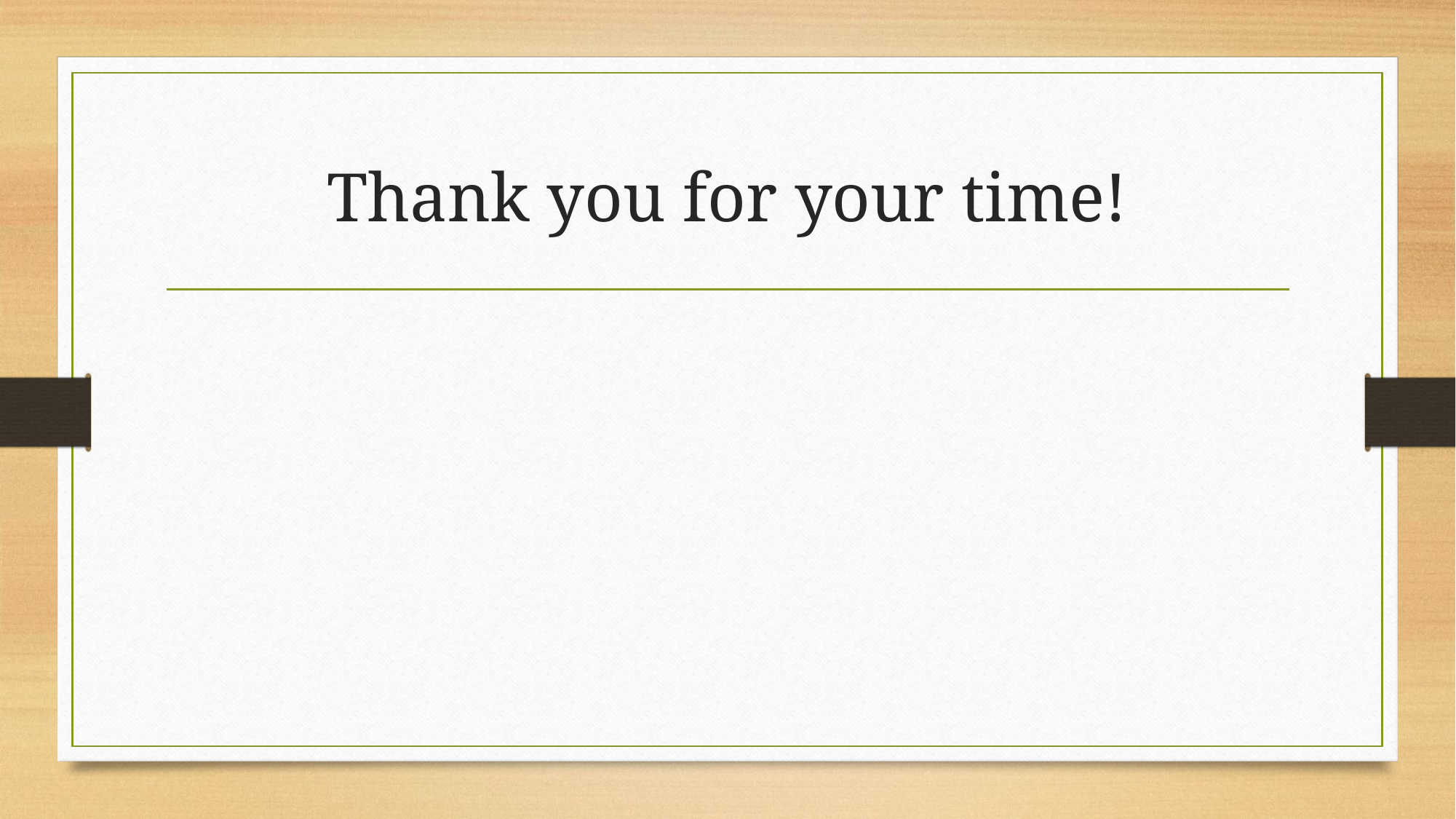

# Thank you for your time!
