# Supplementary material for: Digital health technologies for peripartum depression management among low-socioeconomic populations: perspectives from patients, providers, and social media channels
Source: BMC Pregnancy Childbirth. 2023 Jun 3;23:411. doi: 10.1186/s12884-023-05729-9 (PMC10239590; doi:10.1186/s12884-023-05729-9)
Supplement: Supplementary file 3 — Supplementary Material 3: Appendix C [file 12884_2023_5729_MOESM3_ESM.pptx]

## Slide 1
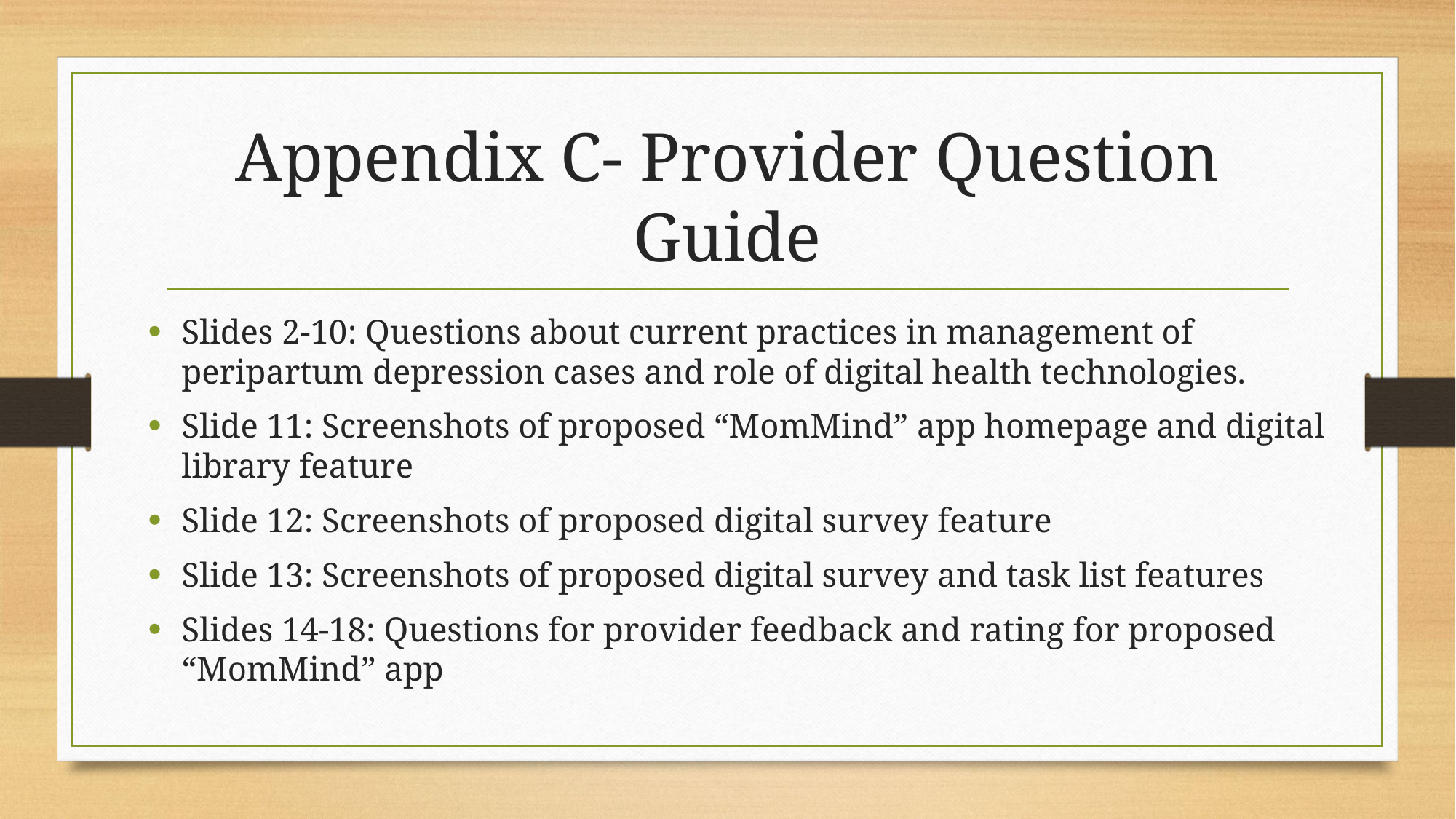

# Appendix C- Provider Question Guide
Slides 2-10: Questions about current practices in management of peripartum depression cases and role of digital health technologies.
Slide 11: Screenshots of proposed “MomMind” app homepage and digital library feature
Slide 12: Screenshots of proposed digital survey feature
Slide 13: Screenshots of proposed digital survey and task list features
Slides 14-18: Questions for provider feedback and rating for proposed “MomMind” app

## Slide 2
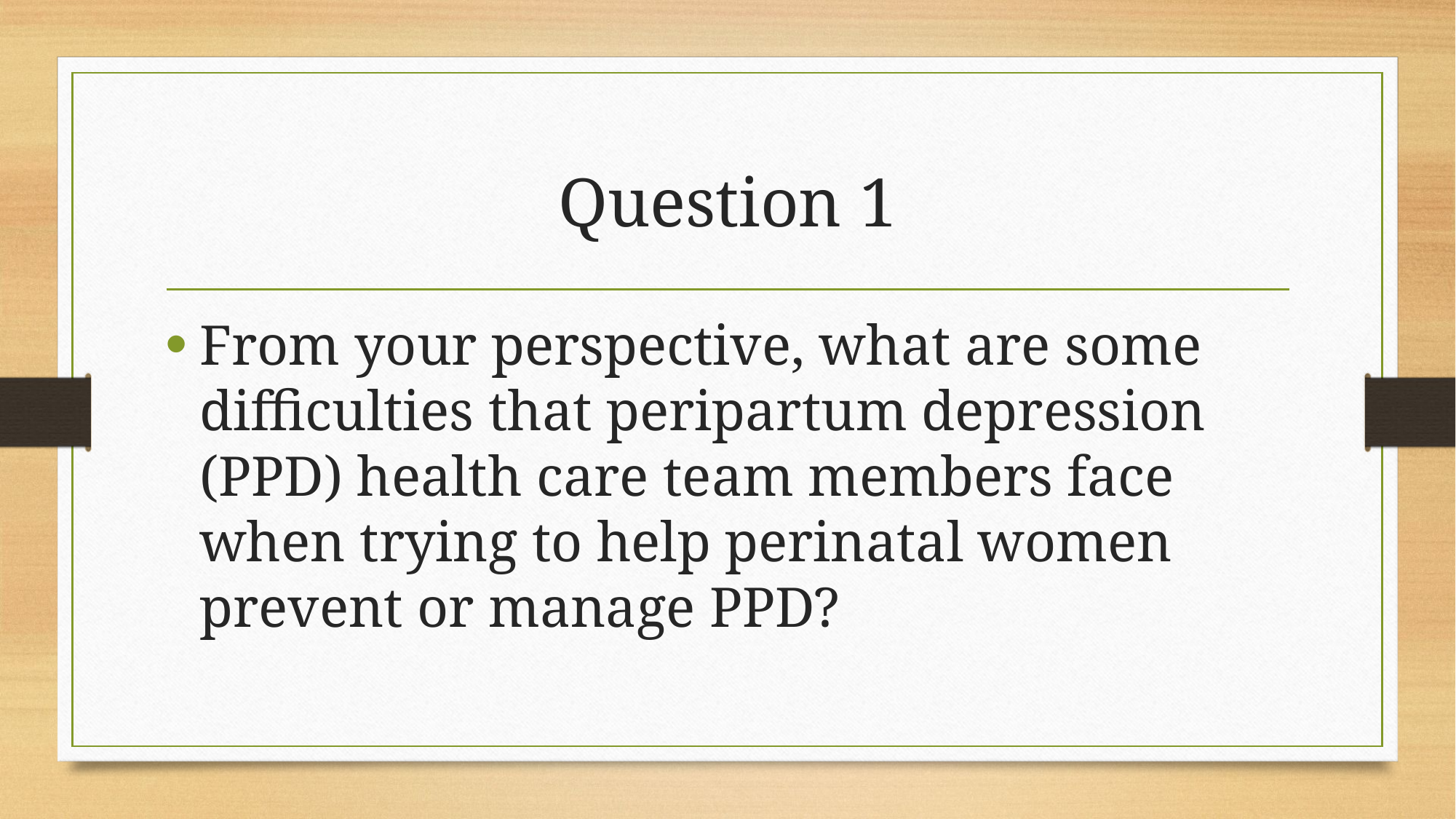

# Question 1
From your perspective, what are some difficulties that peripartum depression (PPD) health care team members face when trying to help perinatal women prevent or manage PPD?

## Slide 3
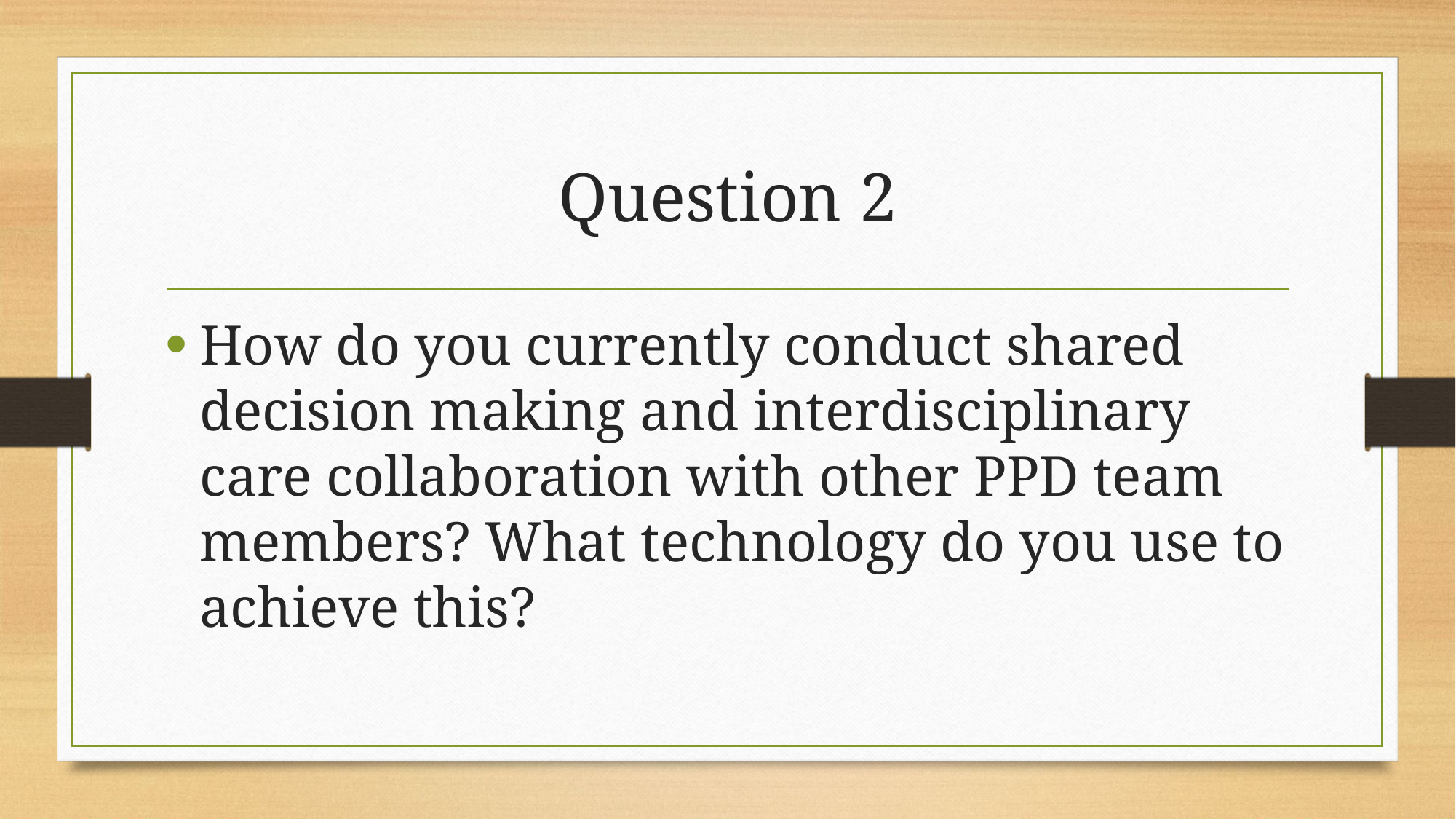

# Question 2
How do you currently conduct shared decision making and interdisciplinary care collaboration with other PPD team members? What technology do you use to achieve this?

## Slide 4
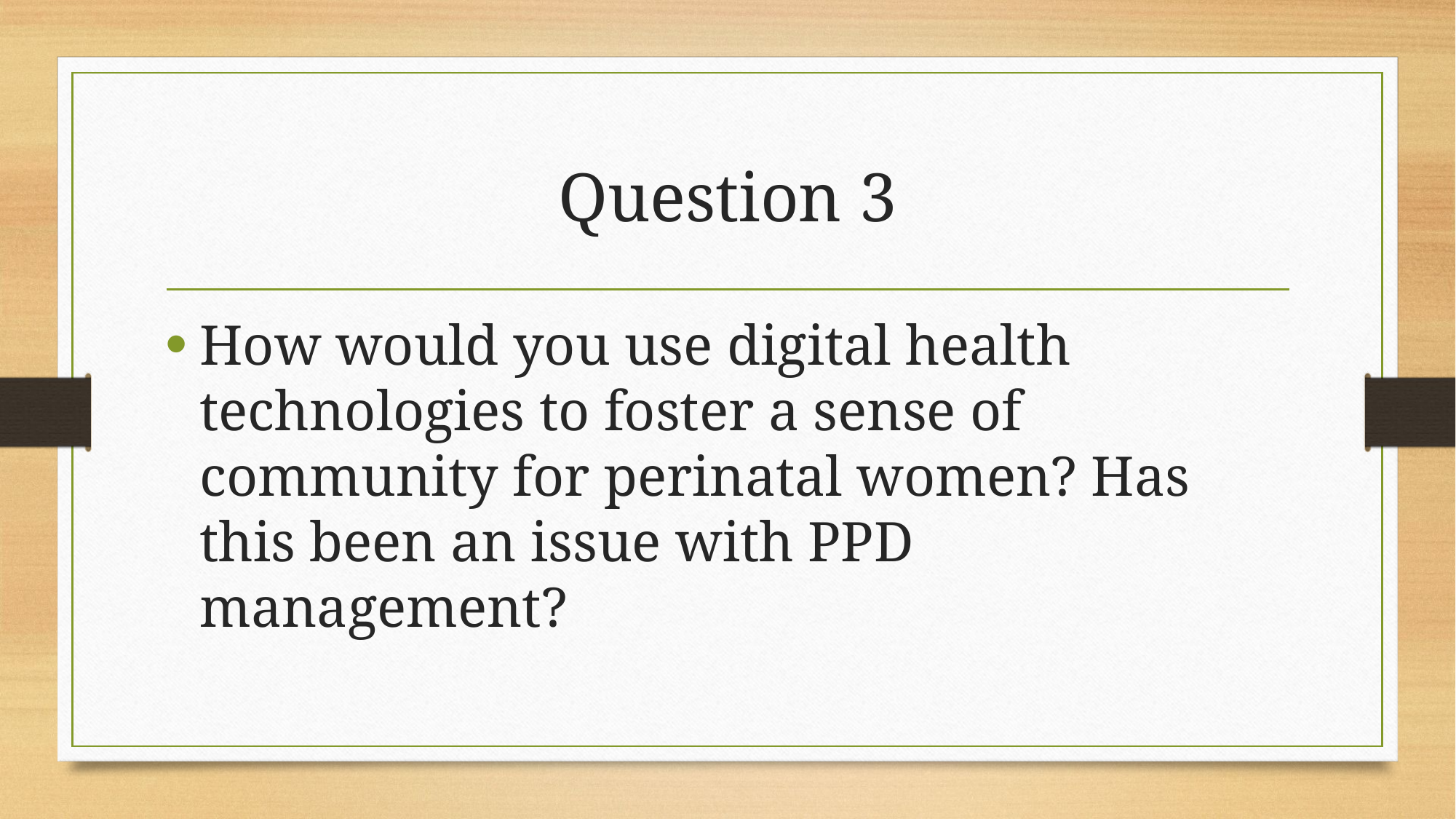

# Question 3
How would you use digital health technologies to foster a sense of community for perinatal women? Has this been an issue with PPD management?

## Slide 5
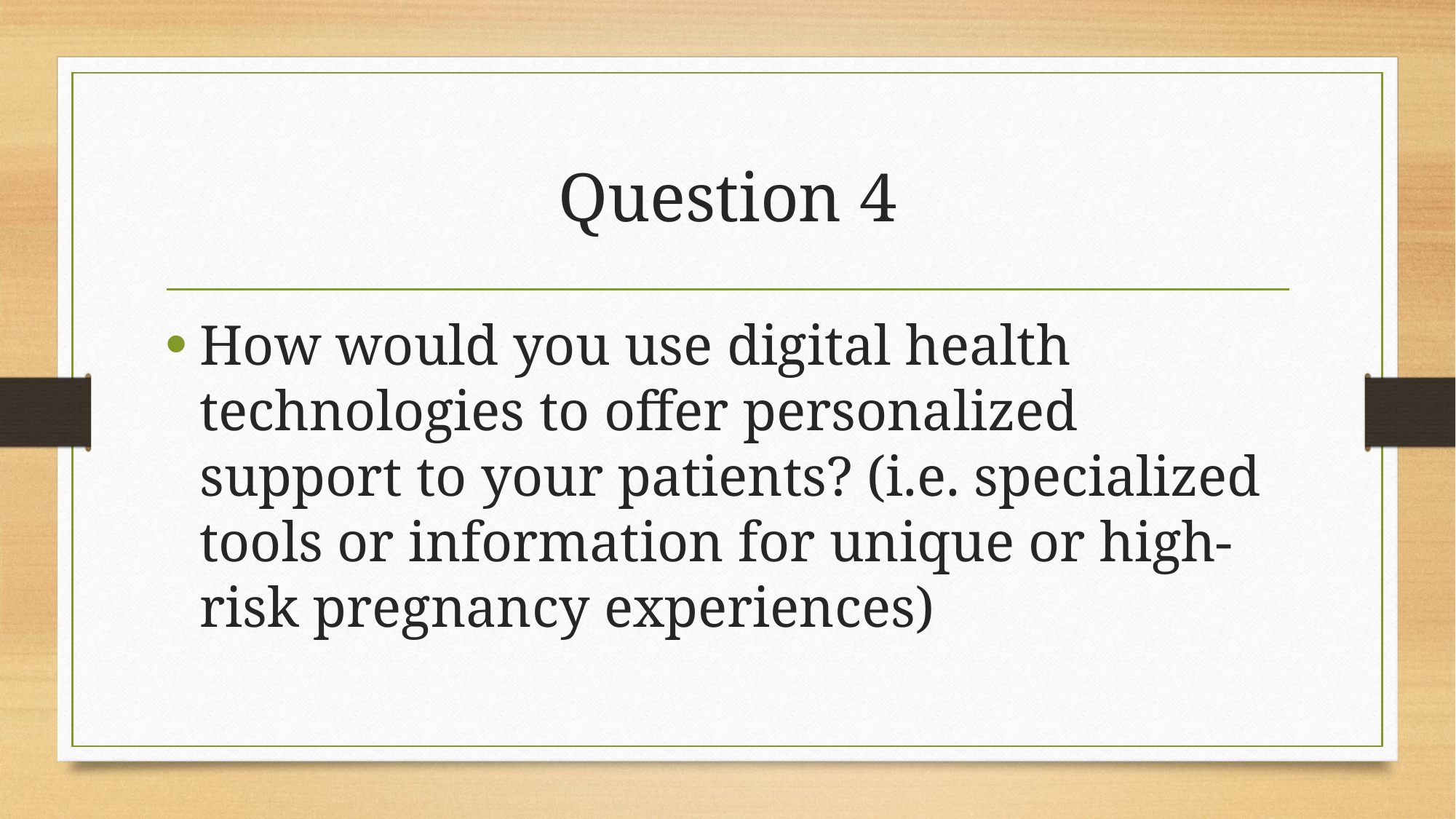

# Question 4
How would you use digital health technologies to offer personalized support to your patients? (i.e. specialized tools or information for unique or high-risk pregnancy experiences)

## Slide 6
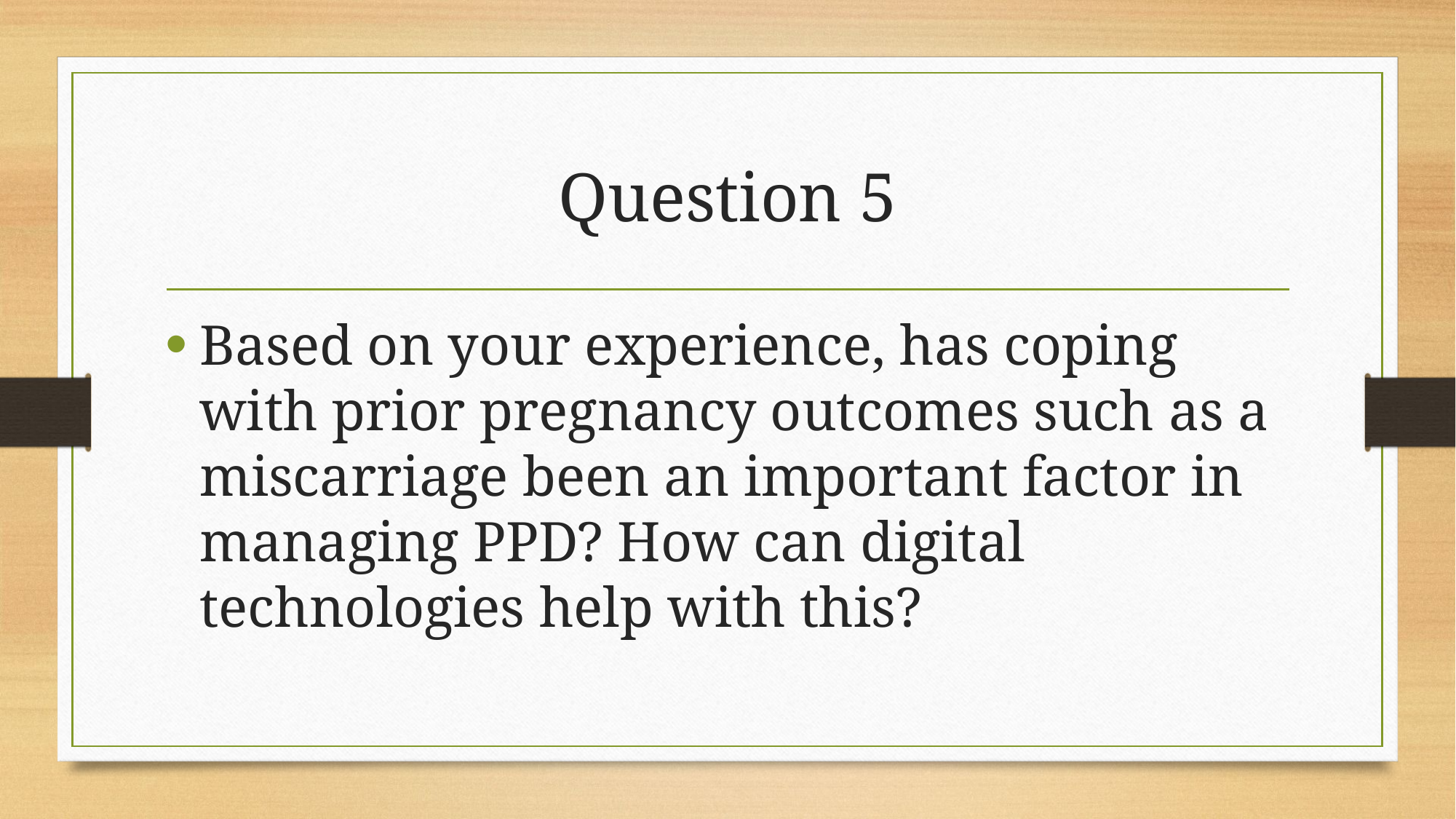

# Question 5
Based on your experience, has coping with prior pregnancy outcomes such as a miscarriage been an important factor in managing PPD? How can digital technologies help with this?

## Slide 7
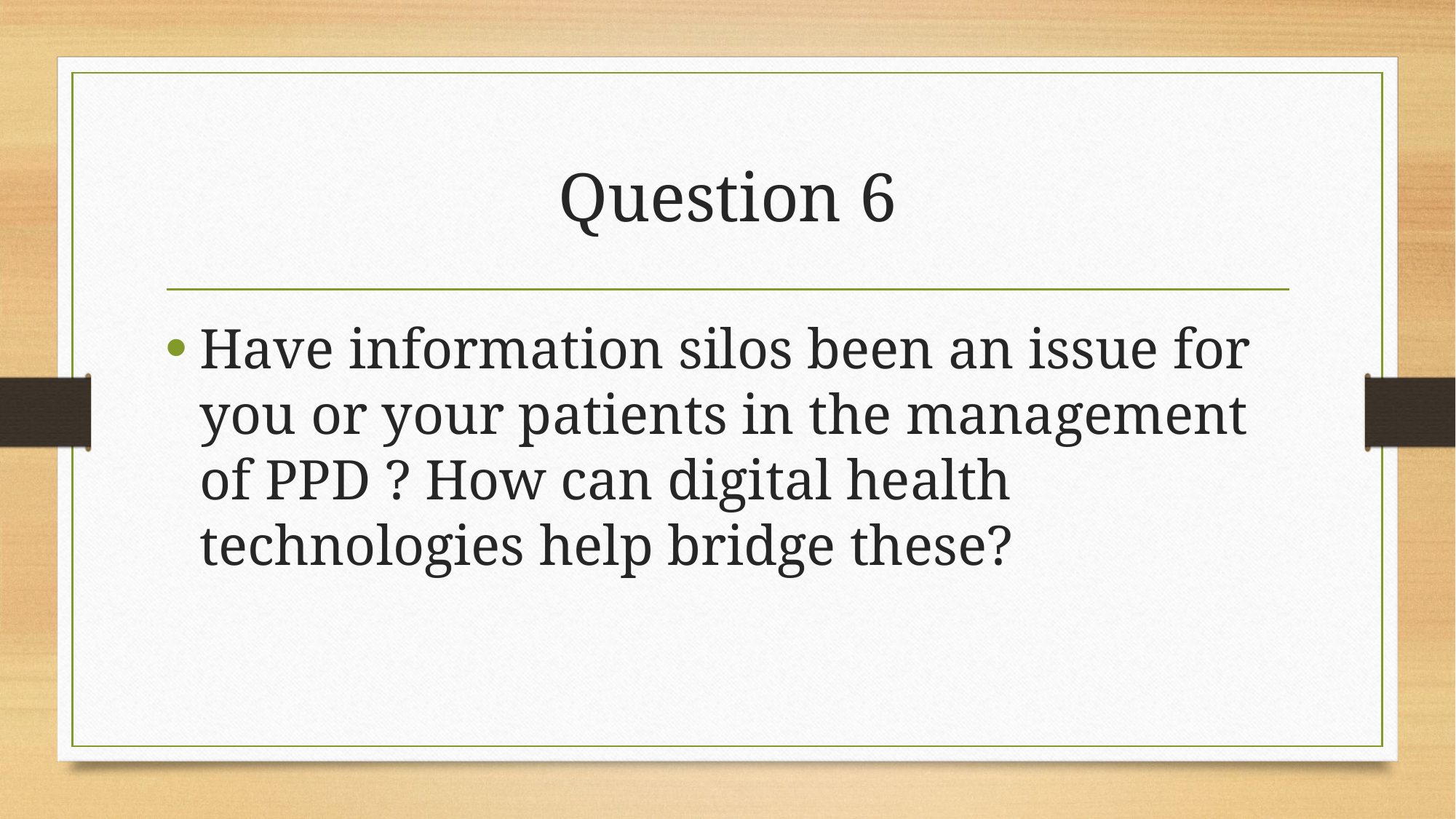

# Question 6
Have information silos been an issue for you or your patients in the management of PPD ? How can digital health technologies help bridge these?

## Slide 8
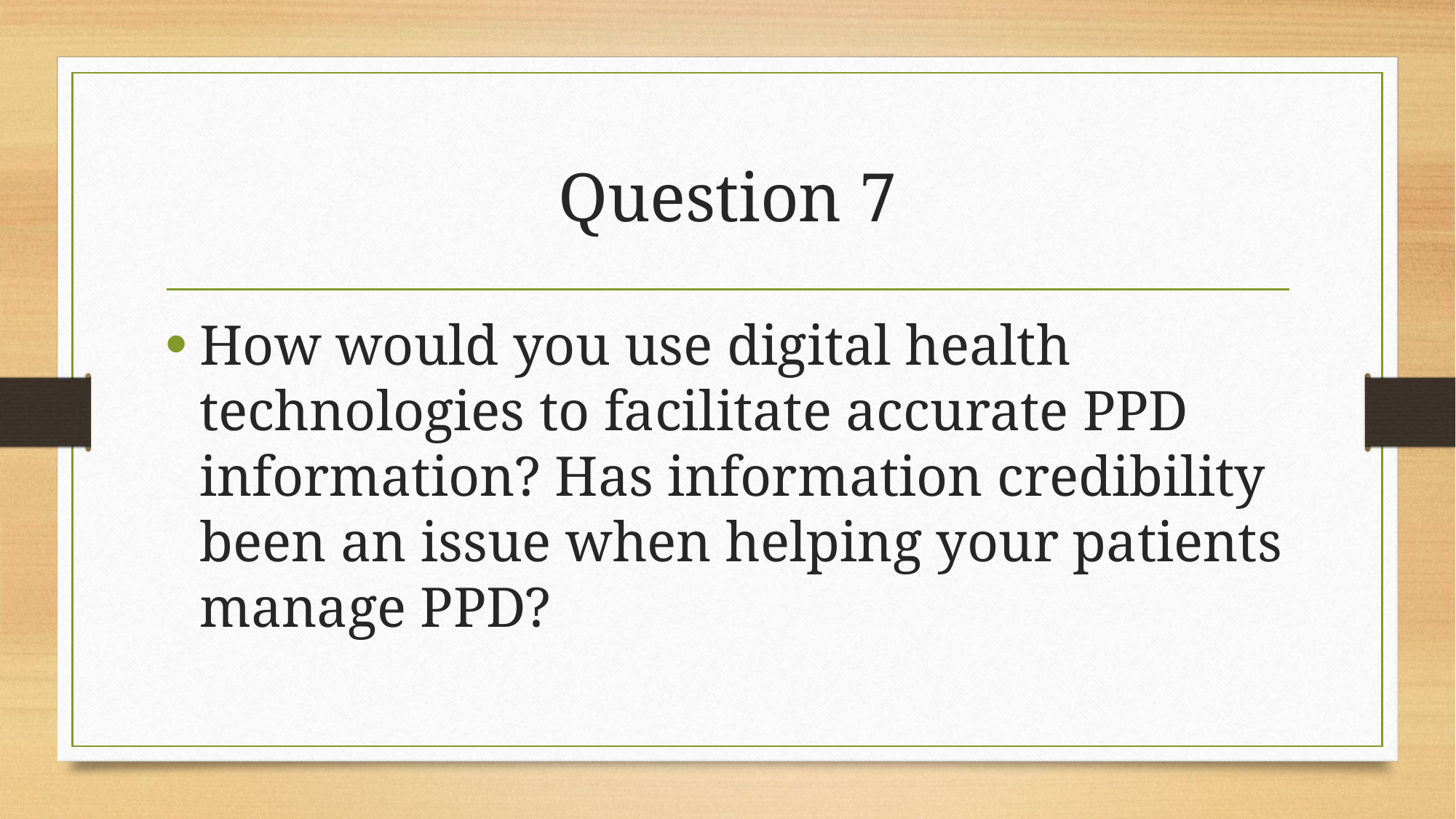

# Question 7
How would you use digital health technologies to facilitate accurate PPD information? Has information credibility been an issue when helping your patients manage PPD?

## Slide 9
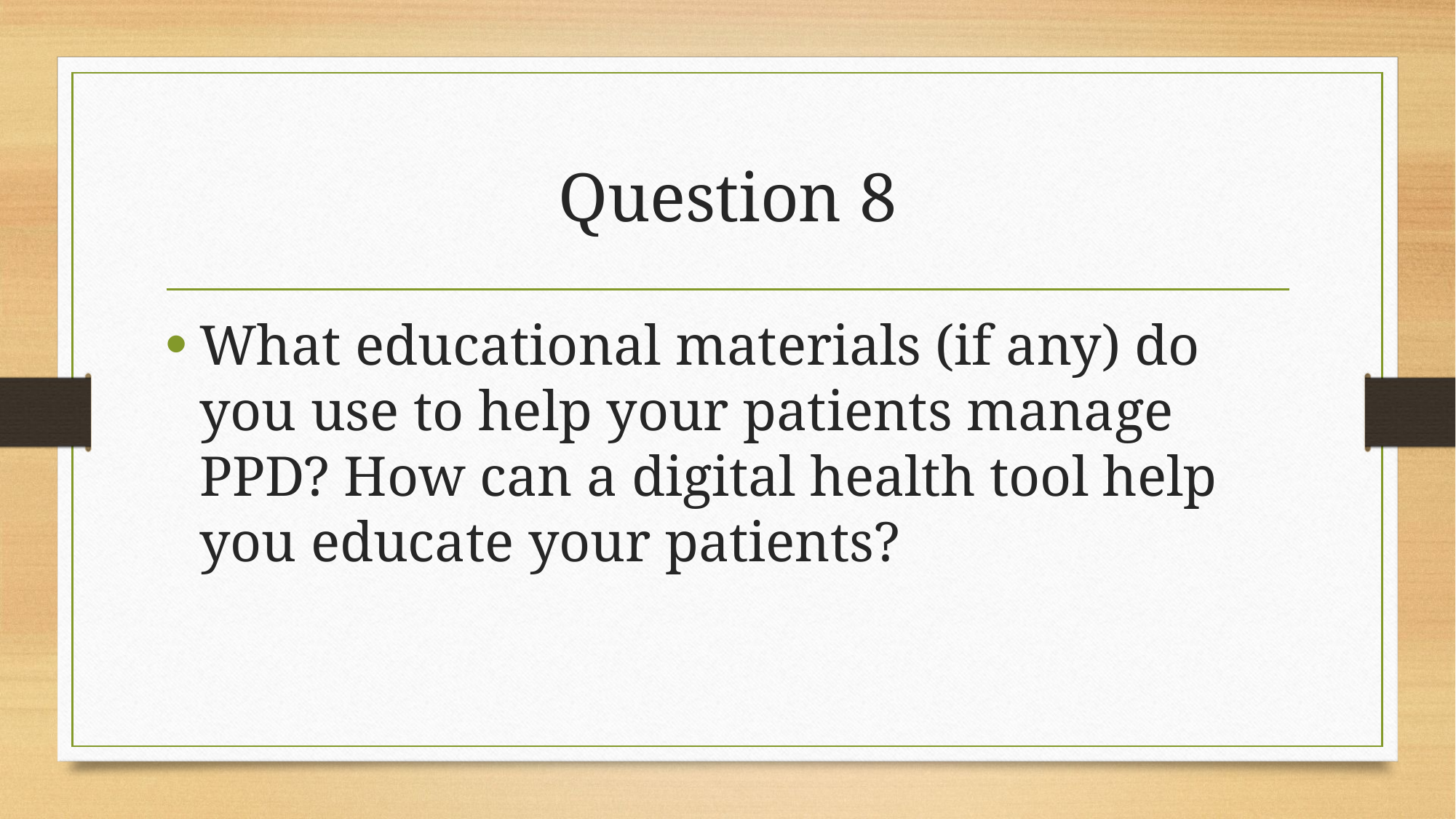

# Question 8
What educational materials (if any) do you use to help your patients manage PPD? How can a digital health tool help you educate your patients?

## Slide 10
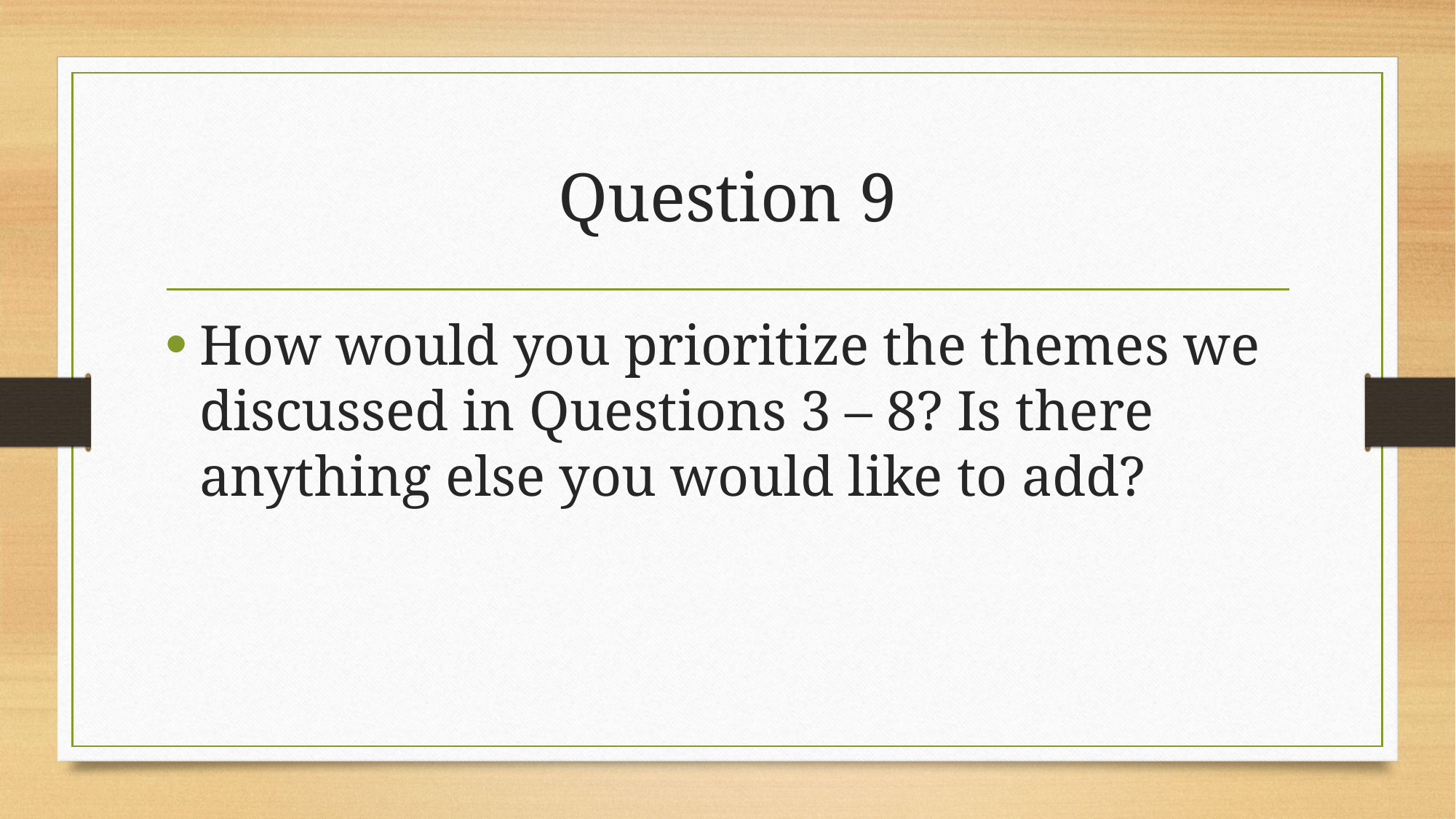

# Question 9
How would you prioritize the themes we discussed in Questions 3 – 8? Is there anything else you would like to add?

## Slide 11
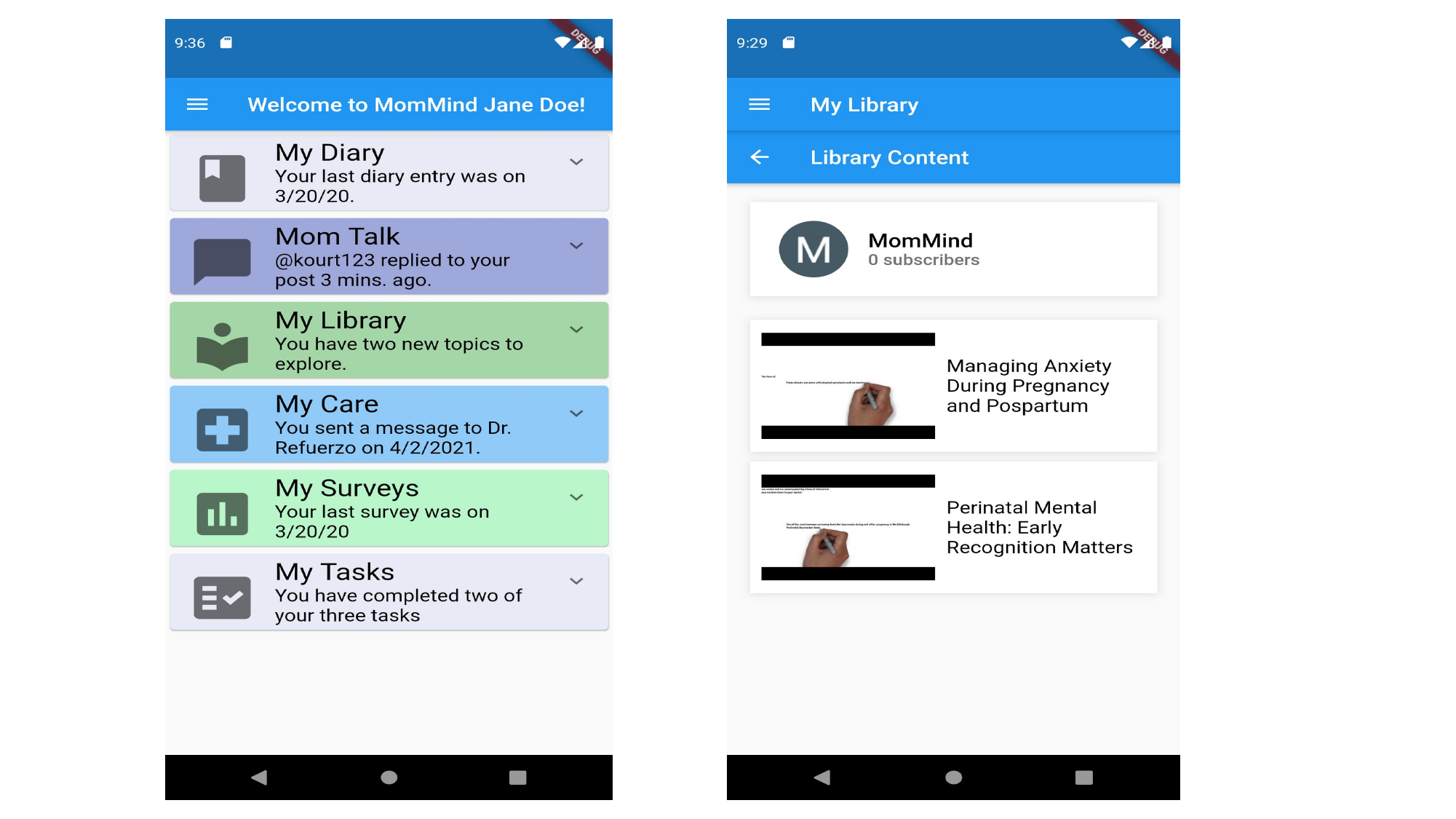

## Slide 12
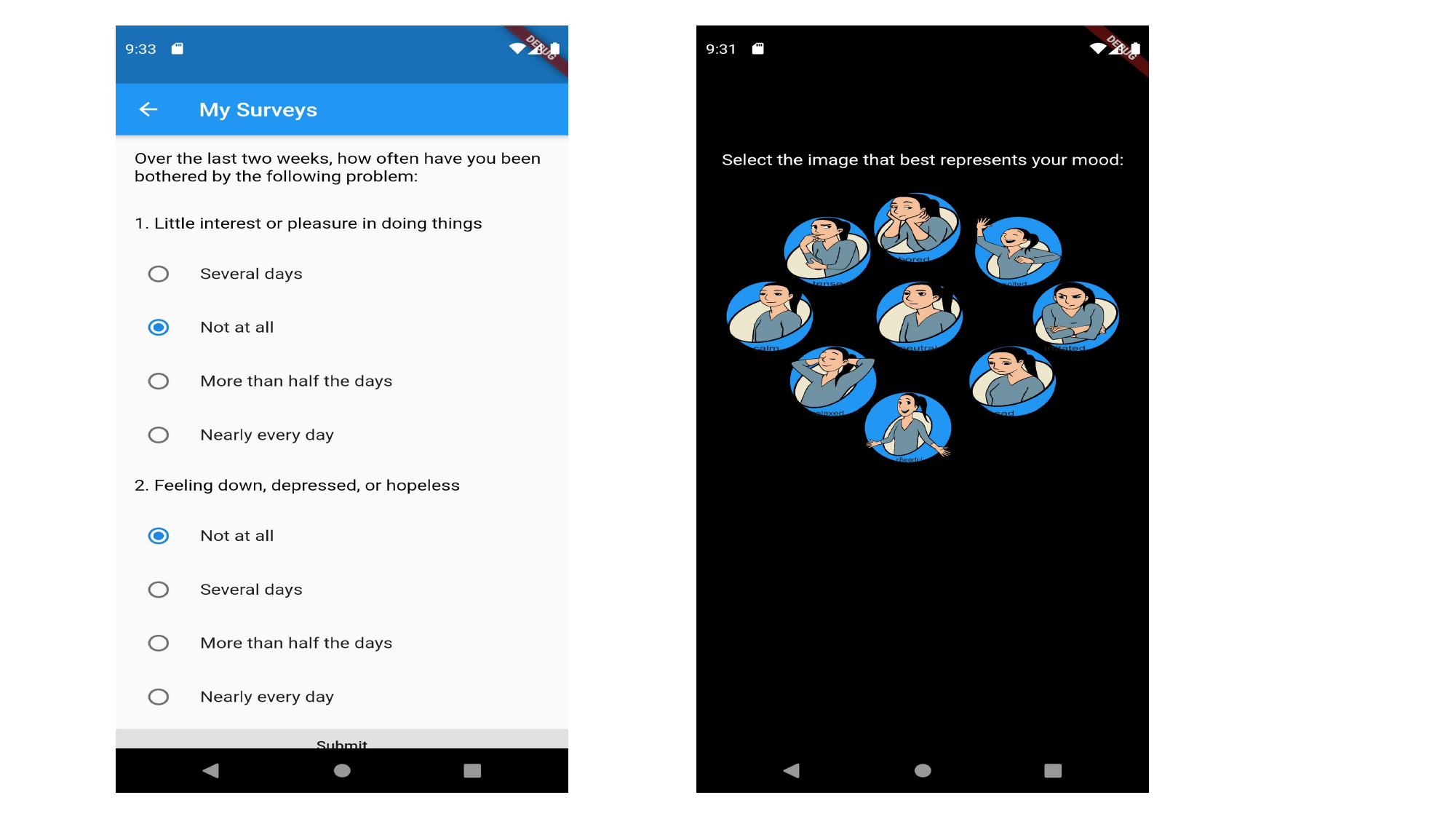

## Slide 13
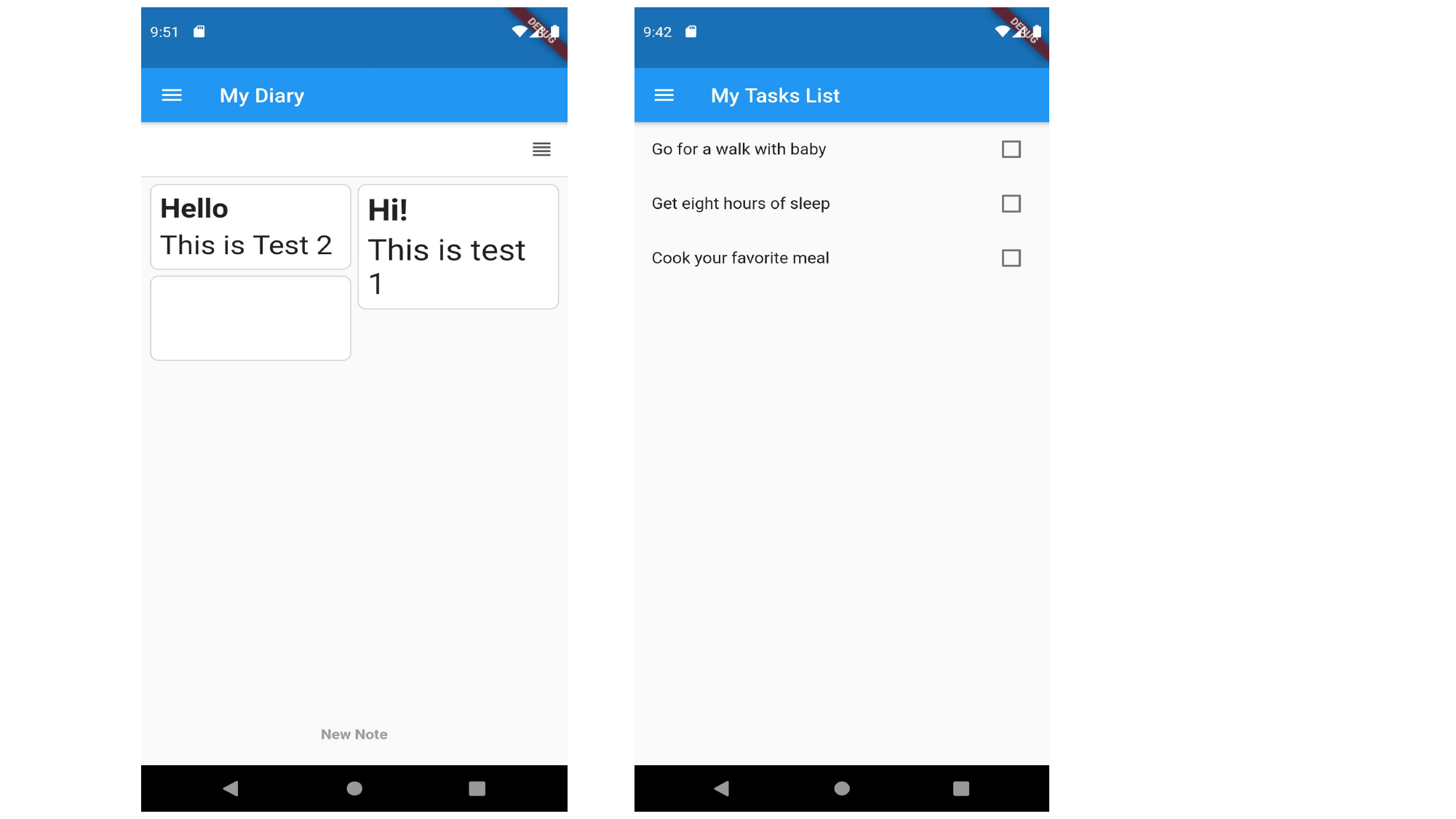

## Slide 14
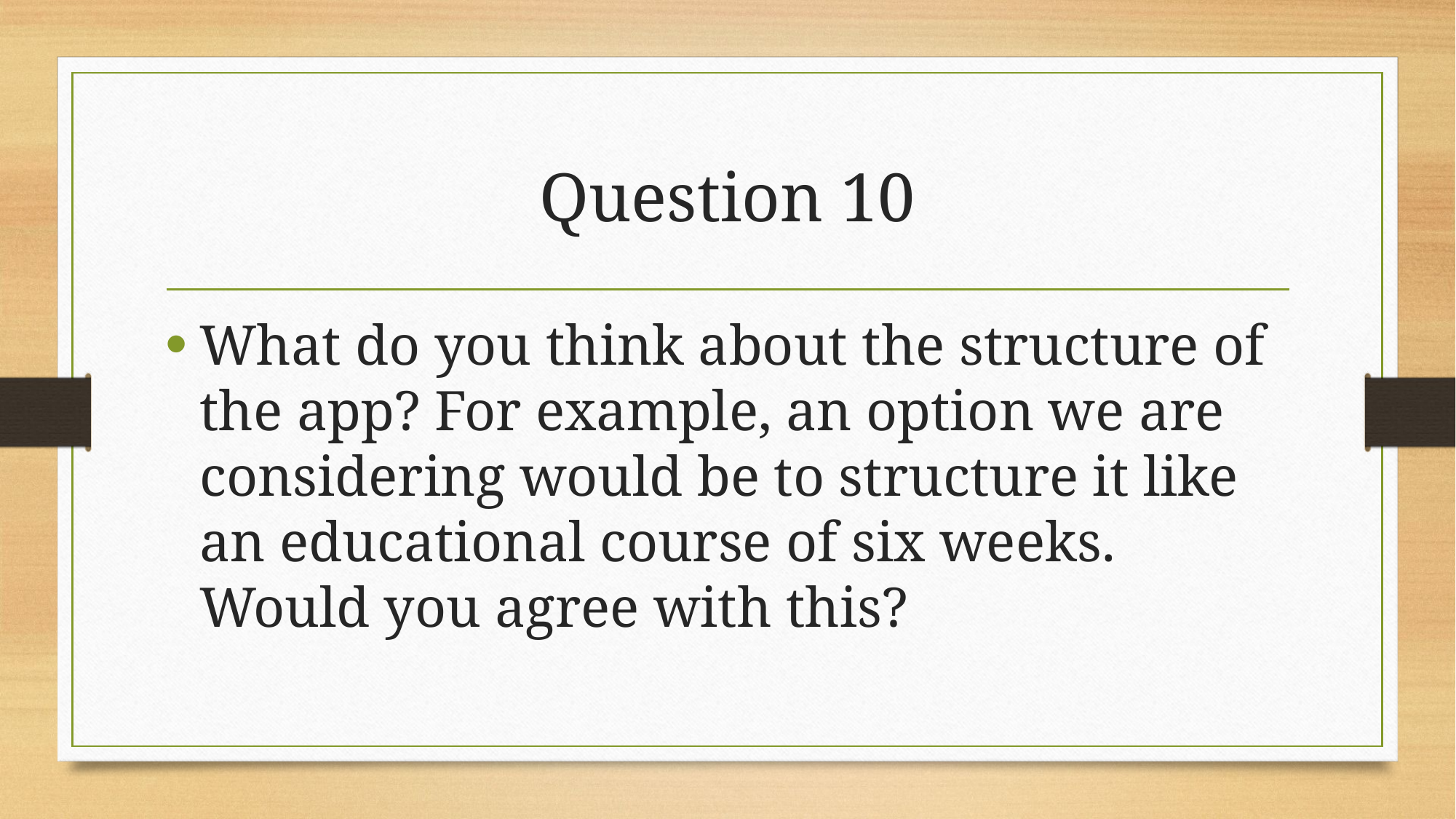

# Question 10
What do you think about the structure of the app? For example, an option we are considering would be to structure it like an educational course of six weeks. Would you agree with this?

## Slide 15
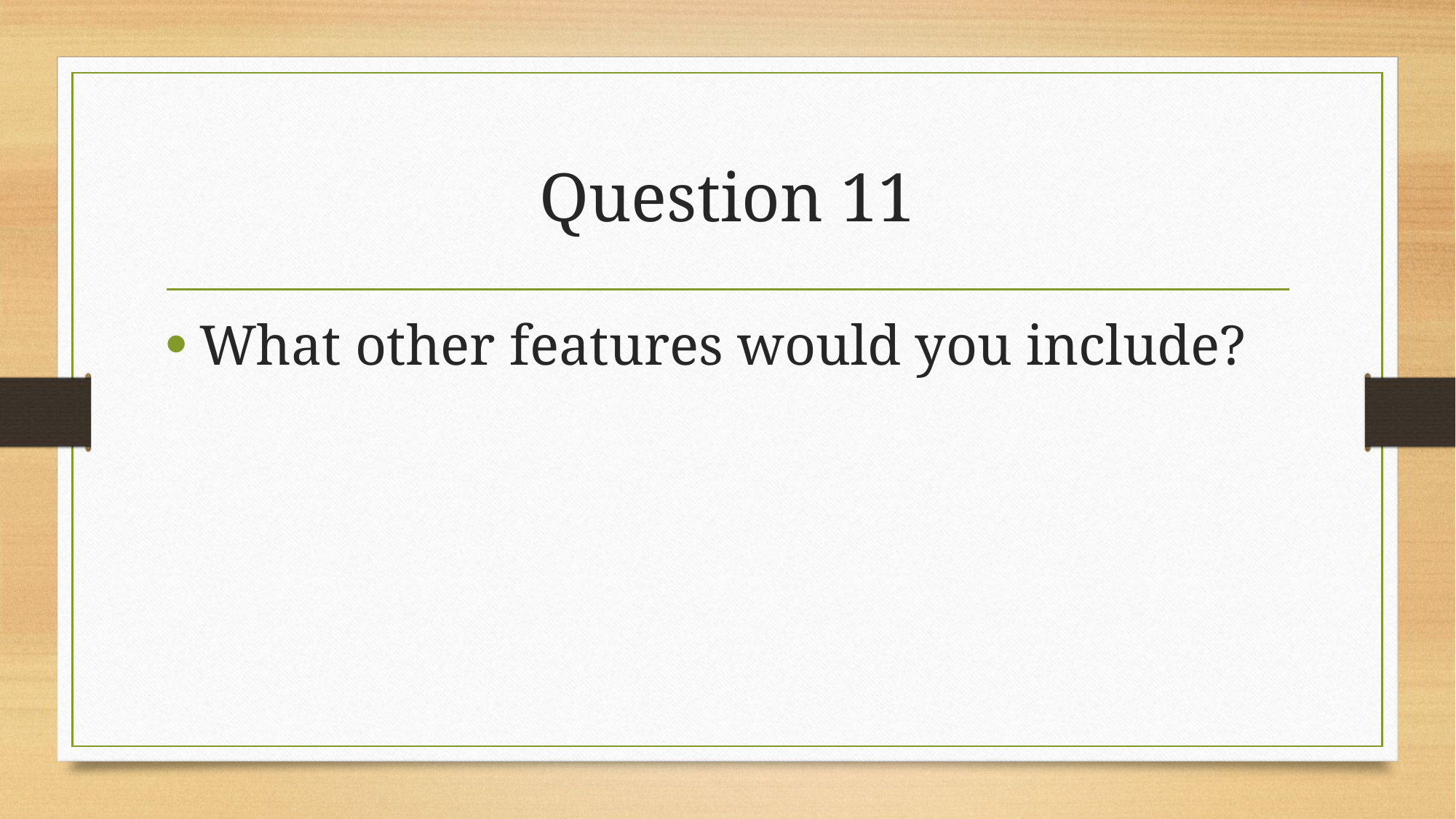

# Question 11
What other features would you include?

## Slide 16
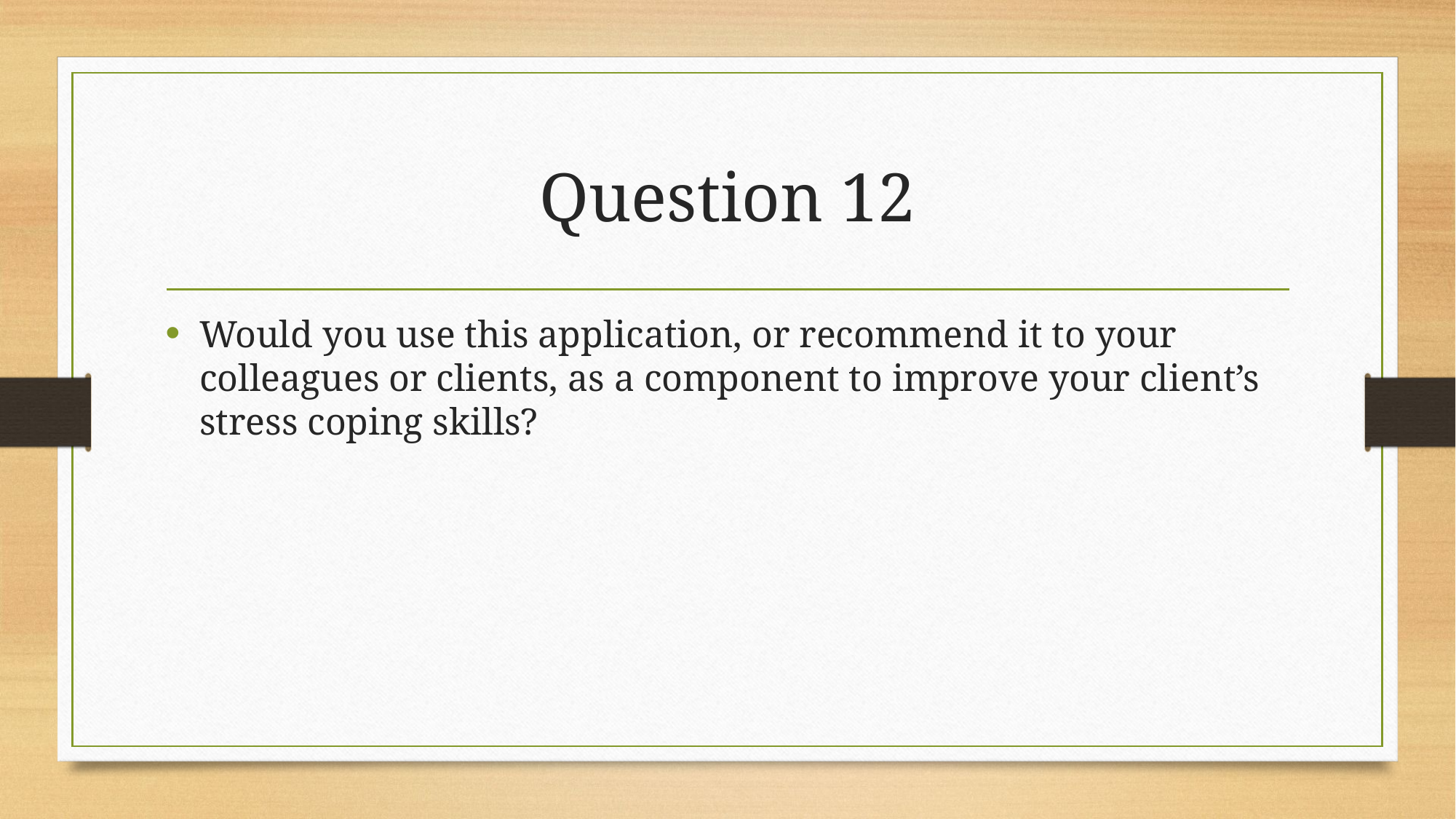

# Question 12
Would you use this application, or recommend it to your colleagues or clients, as a component to improve your client’s stress coping skills?

## Slide 17
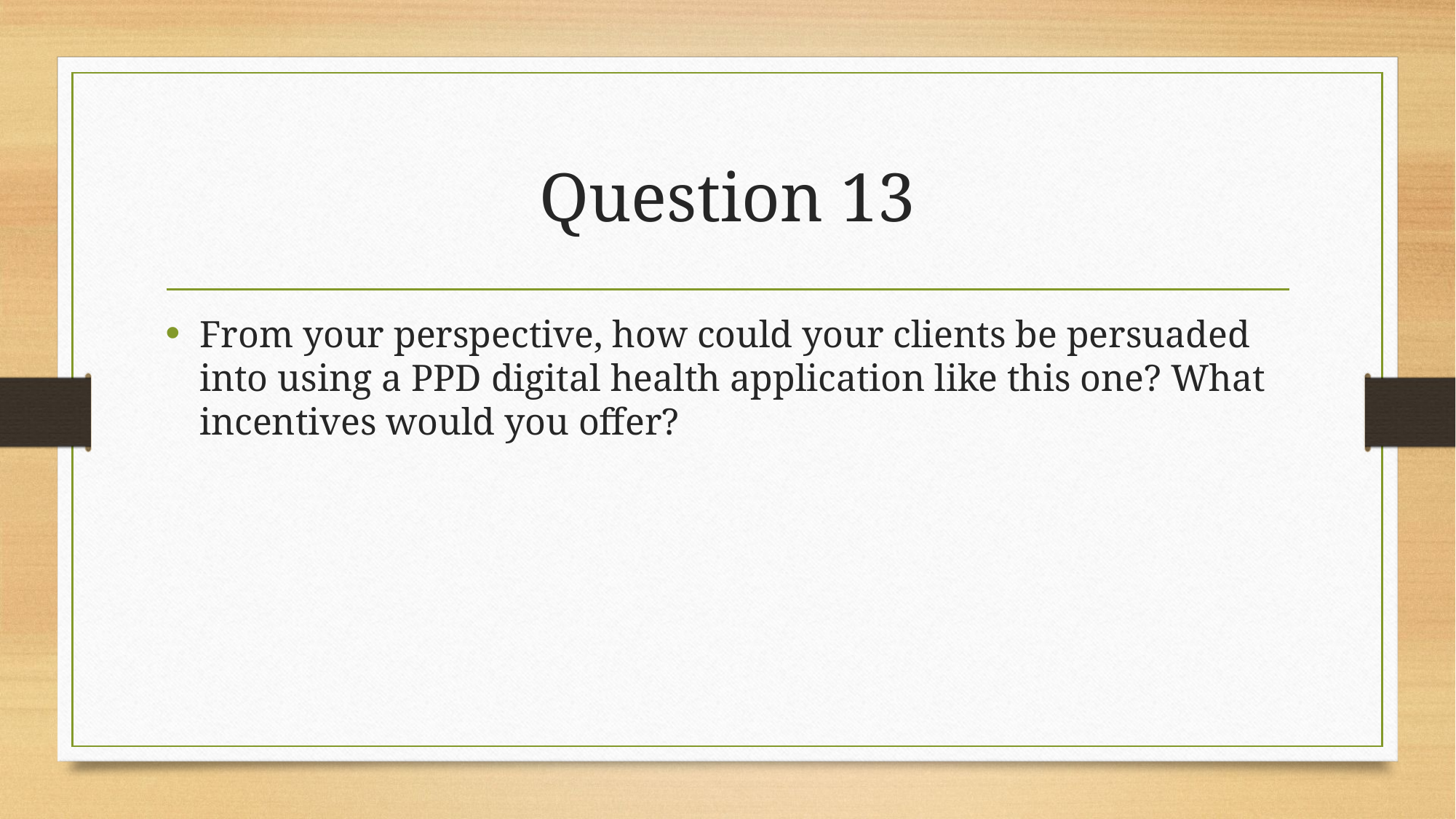

# Question 13
From your perspective, how could your clients be persuaded into using a PPD digital health application like this one? What incentives would you offer?

## Slide 18
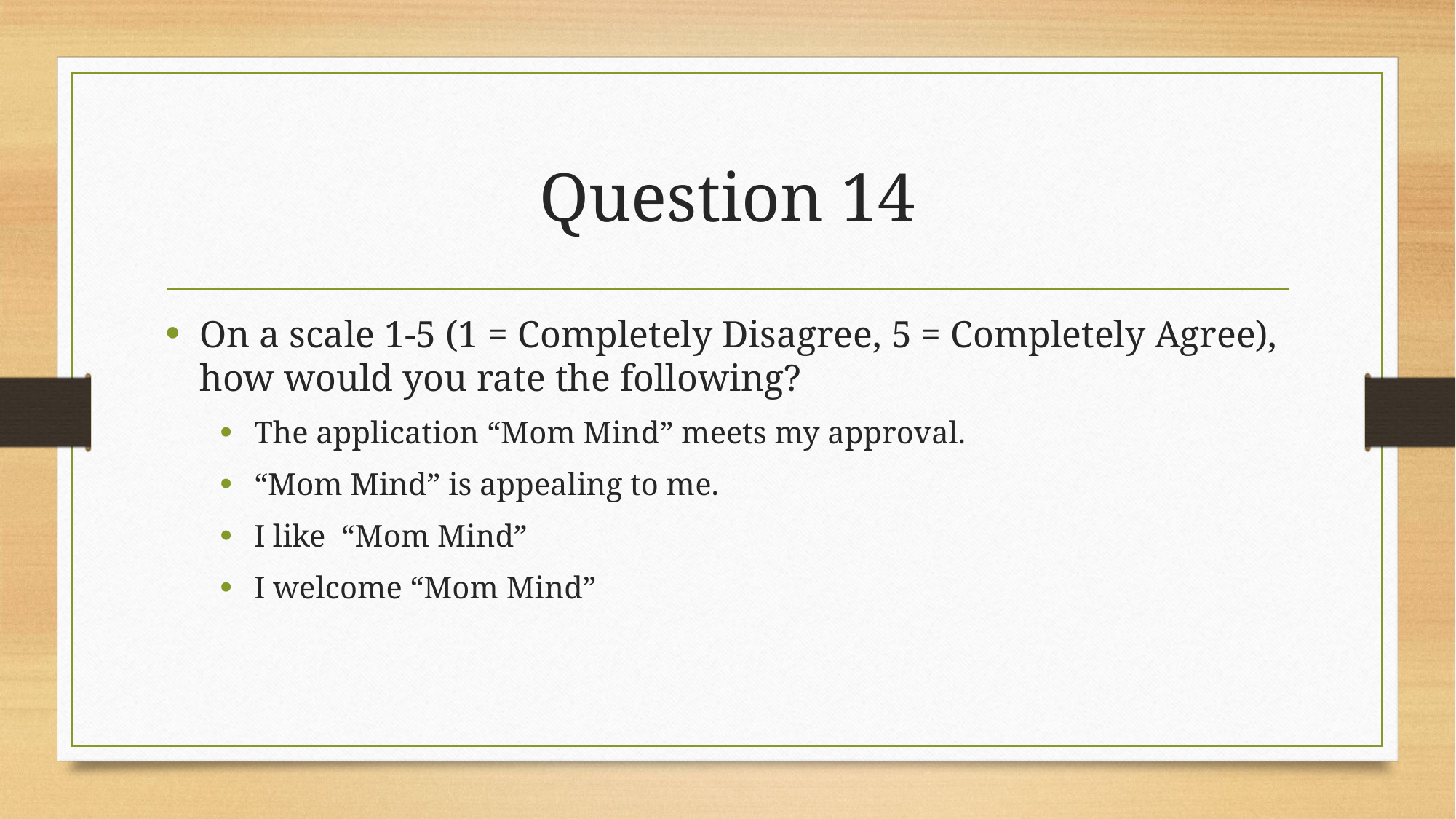

# Question 14
On a scale 1-5 (1 = Completely Disagree, 5 = Completely Agree), how would you rate the following?
The application “Mom Mind” meets my approval.
“Mom Mind” is appealing to me.
I like “Mom Mind”
I welcome “Mom Mind”

## Slide 19
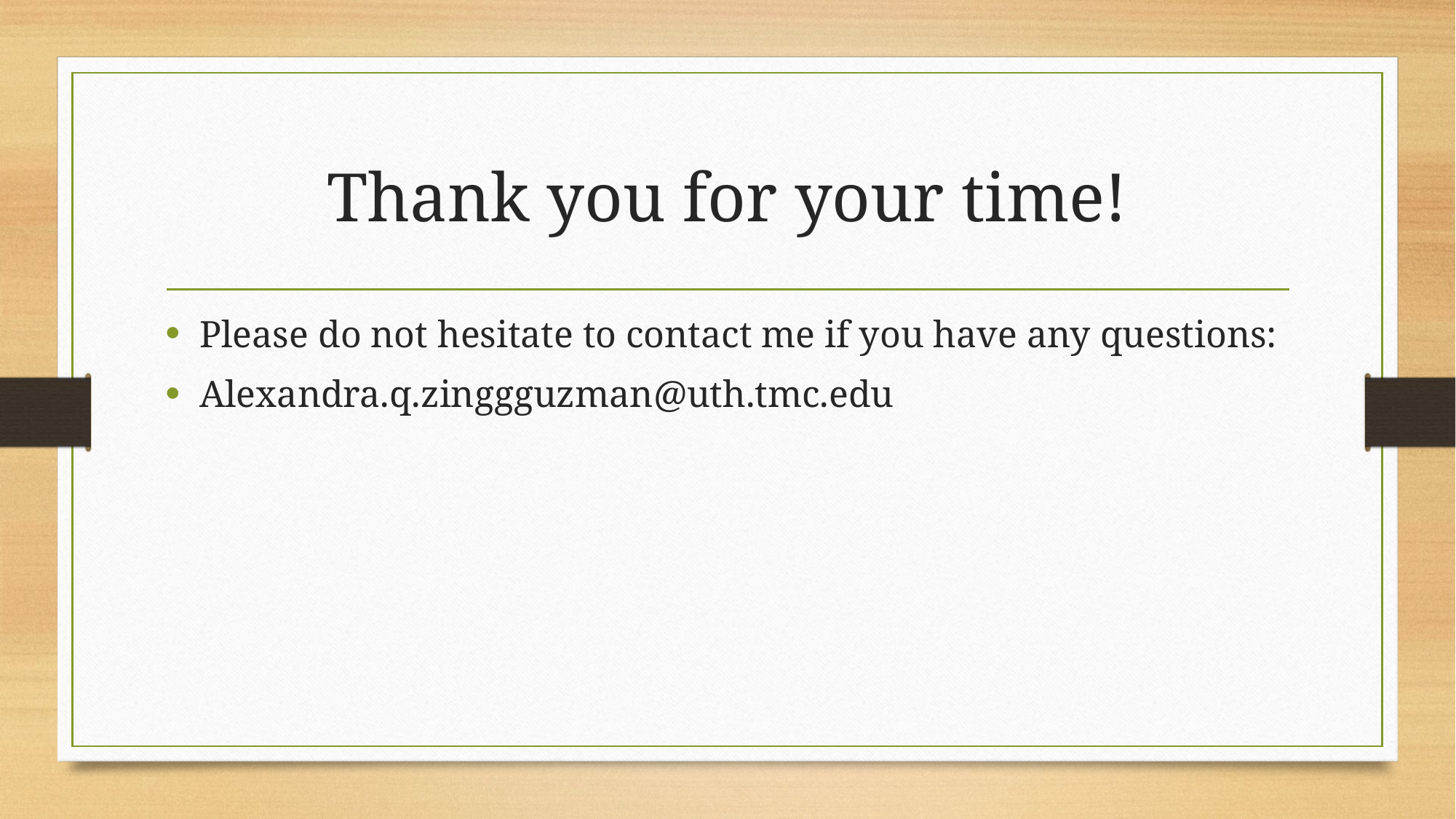

# Thank you for your time!
Please do not hesitate to contact me if you have any questions:
Alexandra.q.zinggguzman@uth.tmc.edu
